# Structural Characterization of N‐Linked Glycans in the Receptor Binding Domain of the SARS‐CoV‐2 Spike Protein and their Interactions with Human Lectins

**DOI:** 10.1002/anie.202011015

**Published:** 2020-10-22

**Authors:** Maria Pia Lenza, Iker Oyenarte, Tammo Diercks, Jon Imanol Quintana, Ana Gimeno, Helena Coelho, Ana Diniz, Francesca Peccati, Sandra Delgado, Alexandre Bosch, Mikel Valle, Oscar Millet, Nicola G. A. Abrescia, Asís Palazón, Filipa Marcelo, Gonzalo Jiménez‐Osés, Jesús Jiménez‐Barbero, Ana Ardá, June Ereño‐Orbea

**Affiliations:** ^1^ CIC bioGUNE Basque Research and Technology Alliance BRTA Bizkaia Technology Park 48162 Derio Spain; ^2^ Ikerbasque, Basque Foundation for Science 48013 Bilbao Spain; ^3^ Centro de Investigación Biomédica en Red de Enfermedades Hepáticas y Digestivas (CIBERehd) Instituto de Salud Carlos III Madrid Spain; ^4^ UCIBIO REQUIMTE Departamento de Química Faculdade de Ciências e Tecnologia Universidade NOVA de Lisboa 2829-516 Caparica Portugal; ^5^ Department of Organic Chemistry II University of the Basque Country UPV/EHU 48940 Leioa Spain

**Keywords:** glycan, lectin, molecular recognition, receptor binding domain, SARS-CoV2

## Abstract

The glycan structures of the receptor binding domain of the SARS‐CoV2 spike glycoprotein expressed in human HEK293F cells have been studied by using NMR. The different possible interacting epitopes have been deeply analysed and characterized, providing evidence of the presence of glycan structures not found in previous MS‐based analyses. The interaction of the RBD ^13^C‐labelled glycans with different human lectins, which are expressed in different organs and tissues that may be affected during the infection process, has also been evaluated by NMR. In particular, ^15^N‐labelled galectins (galectins‐3, ‐7 and ‐8 N‐terminal), Siglecs (Siglec‐8, Siglec‐10), and C‐type lectins (DC‐SIGN, MGL) have been employed. Complementary experiments from the glycoprotein perspective or from the lectin's point of view have permitted to disentangle the specific interacting epitopes in each case. Based on these findings, 3D models of the interacting complexes have been proposed.

## Graphical abstract

Unprecedent structural details of the glycans of the RBD of SARS‐CoV‐2 spike glycoprotein have been revealed by NMR spectroscopy. Unexpected and non‐previously reported glycoepitopes have been detected. The interaction of the RBD glycoprotein with diverse human lectins has been scrutinised by exploiting the NMR signature of the ^13^C‐glycans. Our analysis permitted to identify the corresponding glycan epitopes responsible for the interaction with each lectin.

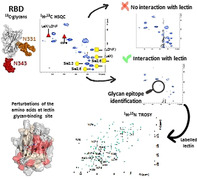

## Introduction

**Figure 1:**
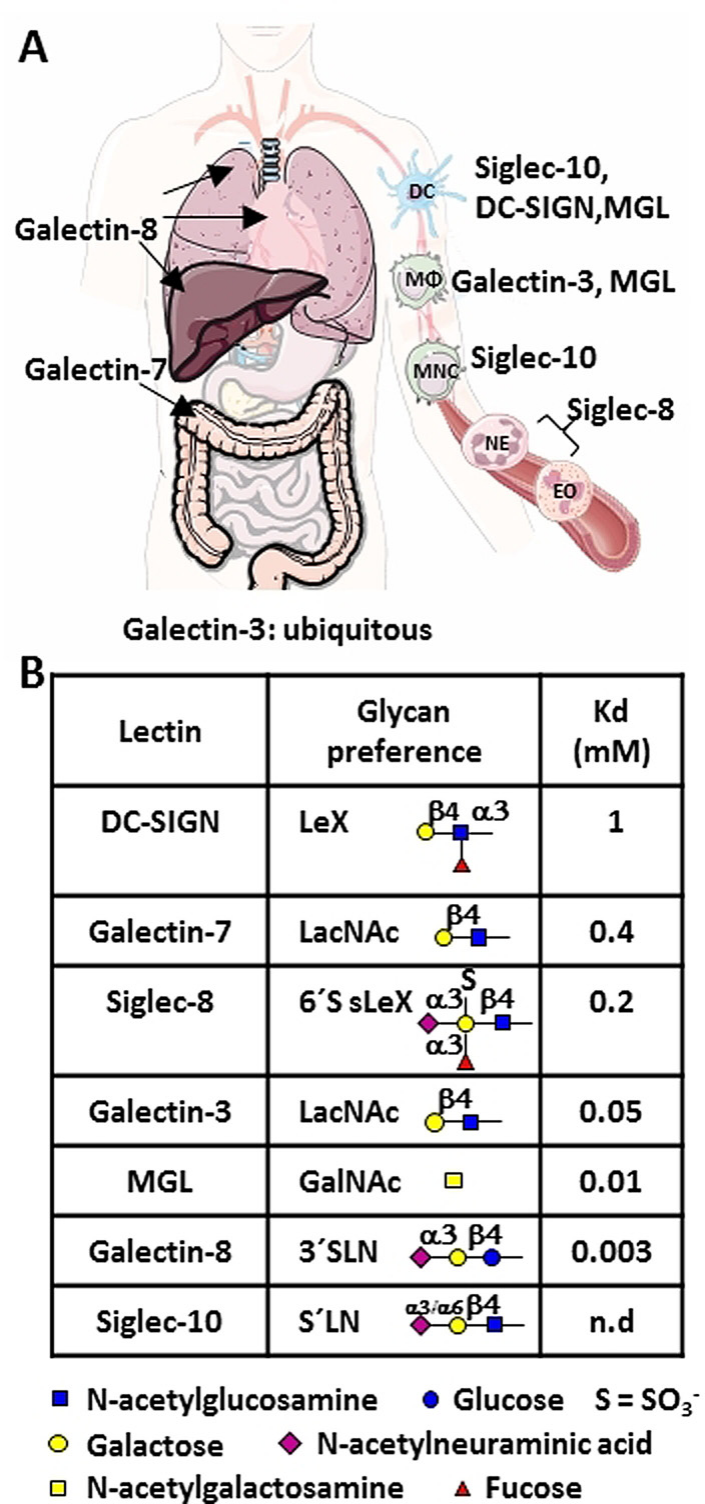
A) Panel of human lectins employed herein along with their locations in human organs and tissues. B) Major glycan specificities and binding affinities for DC‐SIGN,^[20]^ galectin‐7,^[21]^ Siglec‐8,^[22]^ galectin‐3,^[23]^ MGL,^[24]^ galectin‐8^[21]^ and siglec‐10^[25]^ are given. Glycans are represented in SNFG symbols.^[26]^

The current COVID‐19 pandemic caused by SARS‐CoV‐2 coronavirus represents an enormous health and social problem.[^1^, ^2^] The virus employs a glycosylated spike protein (S) to bind the angiotensin‐converting enzyme 2 (ACE2) of the host.[^3^, ^4^] In many viral infections (influenza, Ebola, SARS‐CoV, among others), glycan‐mediated interactions are essential for the initial contact between the virus and the host.[^5^, ^6^, ^7^] In fact, glycans modulate molecular recognition events not only in host‐pathogen recognition or infections, but also in tissue differentiation, cell signalling, immune response, and cancer, besides contributing to proper protein folding.^[8]^ In SARS‐CoV‐2, a receptor binding domain (RBD) has been identified that efficiently binds ACE2. Both ACE2 and the RBD are glycosylated, although the RBD glycans do not seem to be directly involved in the interaction, according to the structural data available so far.[^3^, ^4^, ^9^] Additionally, our immune system contains a variety of glycan‐binding proteins (lectins) that are able to specifically detect and bind diverse glycan‐epitopes, triggering innate responses in a glycan‐dependent manner.[^10^, ^11^] In the SARS‐Cov‐2 context, a recent study^[12]^ has suggested the existence of lectin‐mediated molecular pathways that may contribute to viral infection and immune exacerbation, identifying some lectins that bind to the RBD. From the molecular recognition perspective, unravelling these viral glycan‐host lectin interactions at high resolution represents a tremendous scientific challenge. Since N‐glycosylation is not template‐driven, the hallmark is chemical heterogeneity. Glycoprofile analysis remains technically difficult given the huge range of possible monosaccharide combinations and the different ways they can link to each other. Advances in mass spectrometry (MS) allows achieving a global perspective of the glycoprofile of the target protein. In fact, the glycosylation profile of the spike glycoprotein S has been recently described.[^13^, ^14^, ^15^, ^16^] However, given the need of digestion protocols for MS‐based methods, molecular recognition studies should be carried out with procedures that only minimally alter the test samples. In this context, we have herein applied an NMR protocol^[17]^ to characterize the precise glycan structures of the two N‐linked uniformly ^13^C‐labeled glycans (at N331 and N343) in the domain B of subunit S1 (S^B^) from the RBD (hereinafter referred to as RBD) of SARS‐CoV‐2,[^4^, ^18^] produced in human HEK293F cells.^[19]^ Additionally, we have dissected the glycan‐mediated interactions of RBD with a variety of human lectins, which are expressed in different organs and tissues that may be affected during the infection (Figure 1). For this task, two complementary protocols have been employed. On the one hand, signal changes in the 2D ^1^H,^13^C‐HSQC NMR spectra of the ^13^C‐labeled glycans on the RBD have been monitored upon addition of the lectins. Alternatively, signal perturbations in the ^1^H,^15^N‐TROSY/HSQC spectra of the ^15^N‐labelled lectins in the presence of the RBD have been assessed to provide a complementary view on their specific interactions. Our study provides key structural details on the N‐glycan content on the RBD of SARS‐CoV‐2 spike protein, especially respect to the exposed glyco‐epitopes at the terminal chains, prone to participate in lectin recognition. We have identified the specific glycans in the RBD that are recognized by the corresponding lectin.

## Results and Discussion

### Disentangling the Glycoprofile of the RBD Produced in HEK293F Cells

**Figure 2:**
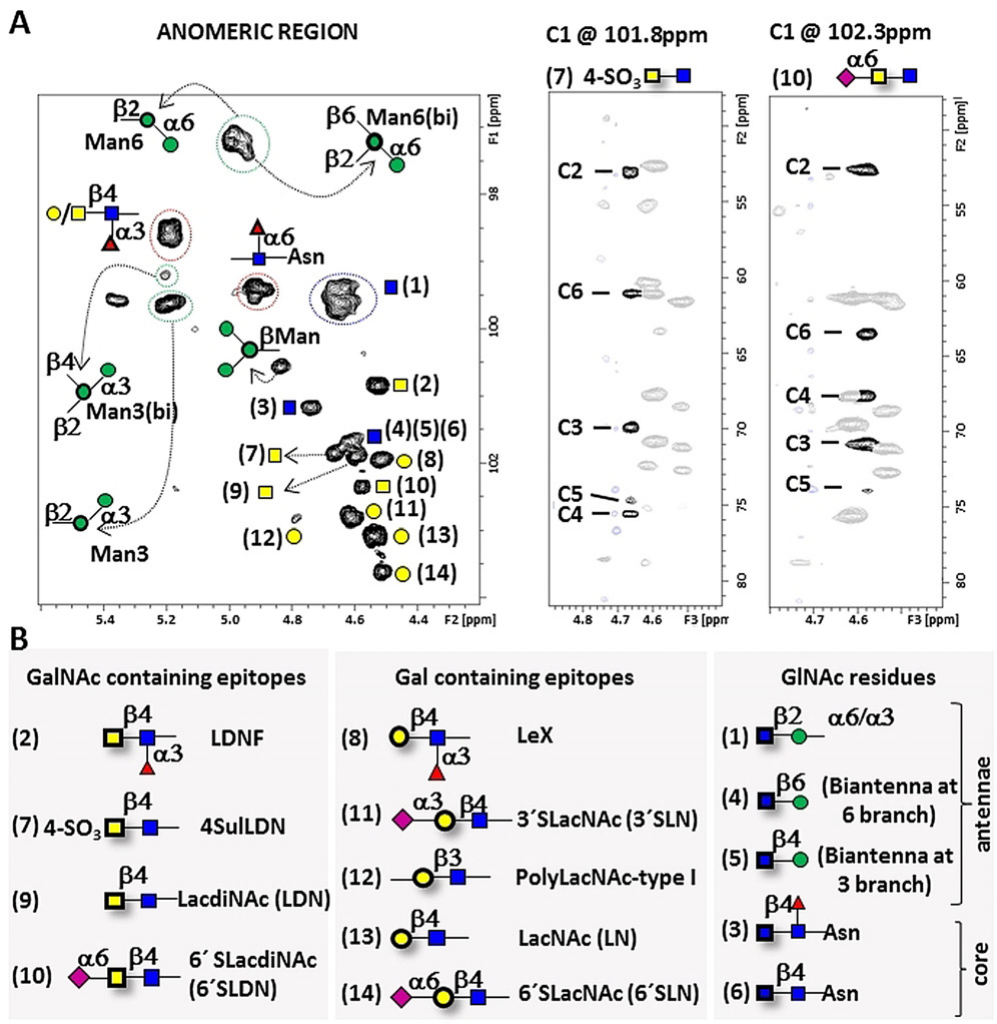
NMR identification of glycan structures on SARS‐CoV‐2 RBD glycoprotein. A) Left: detail of the ^1^H,^13^C‐HSQC of RBD showing the assignment for most anomeric correlations, represented as SNFG symbols.^[26]^ Anomeric correlations for Gal, GalNAc and GlcNAc are identified with a number in brackets. Right: 3D [H′]C′,CH edited HSQC‐TOCSY‐HSQC of RBD, selected planes for C1 GalNAc on the 4SulLDN fragment and for C1 GalNAc on 6′SLDN, showing the correlations to all ^13^C atoms within the pyranose spin system. Nearby cross peaks belonging to other spin systems have been veiled for clarity. B) GalNAc, Gal and GlcNAc containing epitopes in N‐linked glycans on RBD, represented as SNFG symbols.

A NMR‐based approach[^17^, ^27^, ^28^, ^29^, ^30^, ^31^] was employed to perform the glycoprofile analysis of the N‐linked glycans at N331 and N343 at the S^B^ domain of RBD (residues 328‐533). Key regions of the spectra are shown in Figure 2 and Figure S1. In particular, the combined analysis of 3D H′,CH NOESY‐HSQC, H′,CH TOCSY‐HSQC, and H′[C′],CH and [H′]C′,CH edited HSQC‐[^13^C,^13^C]TOCSY‐HSQC (Figure 2) allowed to determine the precise structure of the glycans and their glycosidic linkages (Table S1). The [H′]C′,CH edited HSQC‐TOCSY‐HSQC was instrumental to assign all carbon resonances for every spin system (Figure 2), identifying glycosylated or otherwise chemically modified positions. The presence of terminal N‐acetyllactosamine (LacNAc) units was assessed by the analysis, and appeared also decorated with α2,3‐ (3′SLacNAc or 3′SLN) and α2,6‐linked sialyl (6′SLacNAc or 6′SLN) moieties. The presence of GalNAc‐containing epitopes, β1‐4 linked to GlcNAc, was also evident: terminal GalNAcβ1‐4GlcNAc (LacdiNAc or LDN) was found, along with their α2,6‐sialylated and 4‐O‐sulfated derivatives (6′SLDN and 4SulLDN) that had not been identified in previous MS analyses of the S protein.[^13^, ^14^, ^16^] 4SulLDN was identified due to the exclusive ^1^H/^13^C chemical shifts (Table S1) of position 4 of GalNAc.^[32]^ Another relevant observation was the presence of a high degree of fucosylation, both at the core and at terminal positions, corresponding to LewisX (LeX) and fucosylated LDN (LDNF). The presence of this last epitope was somehow unexpected as it has usually been related to parasites, and is thought to cause immunogenic response in humans.[^33^, ^34^, ^35^] With respect to the N‐glycan architecture, although a quantitative analysis is out of the scope of this study, biantennary complex N‐glycans are the prevalent scaffolds. Signals corresponding to high‐mannose‐type N‐glycans display almost undetectable intensity, while the presence of core bisecting GlcNAc was discarded due to the absence of its characteristic signals (downfield shift of the H4‐C4 correlation for βMan).^[36]^ On the other hand, a minor degree of additional branching to give tri‐ and tetra‐antennae was also verified. The branching at the α3‐antenna occurs through further β1‐4‐linked GlcNAc glycosylation of αMan3, whose anomeric carbon becomes upfield shifted (Figure 2).^[36]^ The branching at the α6 antenna is produced by a GlcNAc β1‐6 linkage to αMan6, whose anomeric correlation can be now distinguished.^[36]^ Additionally, the low intensity of the signals corresponding to the anomeric positions of βGal moieties in type I poly‐LacNAc structures, strongly suggested the absence of these elongations.

Protein N‐glycosylation is a highly complex and tightly regulated event, hitherto not fully understood.[^37^, ^38^] The use of glycoproteins as therapeutics has fostered the development of novel methods to control glycosylation, with special focus on producer‐cell lines, which strongly influence the N‐glycosylation outcome.^[39]^ Different MS‐based studies on the glycosylation pattern of the spike protein in HEK293/F cells have been recently presented.[^13^, ^14^, ^15^, ^16^] However, despite employing the same expression system, these studies yielded slightly different results, which could be partially explained by the use of diverse S proteins (either the trimeric form or the separate S1 and S2 subunits). Indeed, the specific protein structure has been proposed as one of the multiple factors influencing protein glycosylation.[^40^, ^41^] Our results for the RBD of the S protein, which contains two glycosylation sites at N331 and N343, show very important levels of fucosylation (Fuc) and N‐acetyl galactosylation (GalNAc), also reported in some of the MS‐based studies.[^13^, ^14^, ^15^, ^16^] However, our NMR methodology allows defining the precise chemical nature and structural details (glycosidic linkages, sulfation) of the epitopes in which these residues (Fuc, GalNAc) are found. Indeed, glycan motifs not described earlier, as 4SulLDN, 6′SLDN, LeX, and LDNF were evident by NMR.

These moieties, along with terminal LacNAc, LDN, 3′SLN and 6′SLN fragments are predominant epitopes on the outer chains of the RBD N‐glycans. These unexpected findings prompted us to produce a different ^13^C‐labelled glycoprotein, the α subunit of the human high‐affinity Fc receptor for IgE (Fc*ϵ*RIα),[^17^, ^42^] using exactly the same conditions used for the RBD. Interestingly, the superimposition of the ^1^H,^13^C‐HSQC spectra of the glycans of RBD and Fc*ϵ*RIα evidenced the lack of all GalNAc‐containing cross peaks as well as fucosylated LDNF and LeX signals in Fc*ϵ*RIα, as previously observed by employing other conditions (Figure S2). Among other factors, these results suggest that the precise protein structure could influence the glycosylation pattern.[^40^, ^41^, ^43^, ^44^, ^45^] Nevertheless, the NMR methodology described herein allows detecting key features of the epitopes, as sulfation, rather difficult to detect by the potent MS approach.[^46^, ^47^, ^48^]

### Molecular Recognition Studies: The Interaction with Lectins

**Figure 3:**
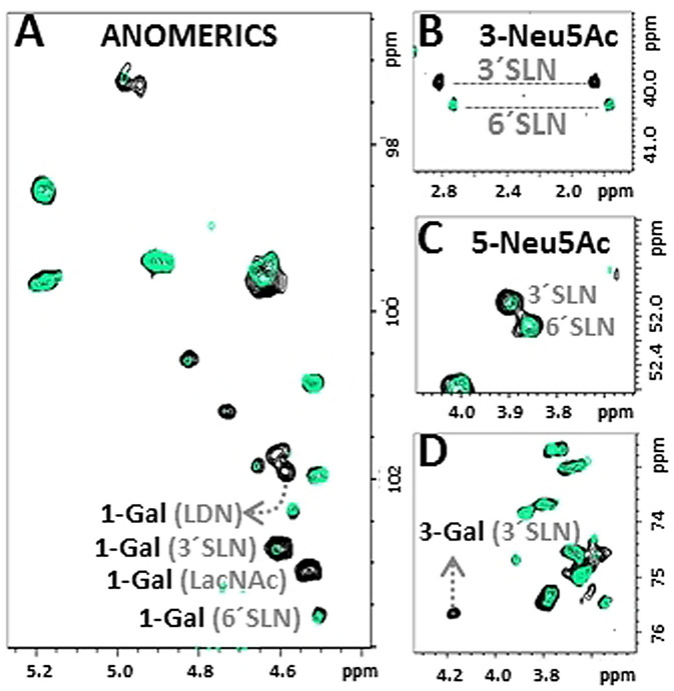
NMR identification of glycan epitopes on the RBD recognized by galectin‐3. Different regions of the ^1^H,^13^C‐HSQC spectrum of RBD alone (in black) and in the presence of 1 equivalent of galectin‐3 (in green). A) Anomeric region: signals for terminal epitopes mostly affected by the presence of galectin‐3 are annotated. B and C) Regions of the H3‐C3 and H5‐C5 (respectively) correlations of terminal Neu5Ac residues: signals for α2‐3 linked residues completely disappear, while those for the α2‐6 linked are barely affected. D) Region showing the signal of H3‐C3 Gal in 3′SLN epitope. The graphical bar representation for the % of volume reduction of selected cross peaks on ^1^H,^13^C‐HSQC of RBD upon addition of galectin‐3 is given in Figure 4 upper panel.

**Figure 4:**
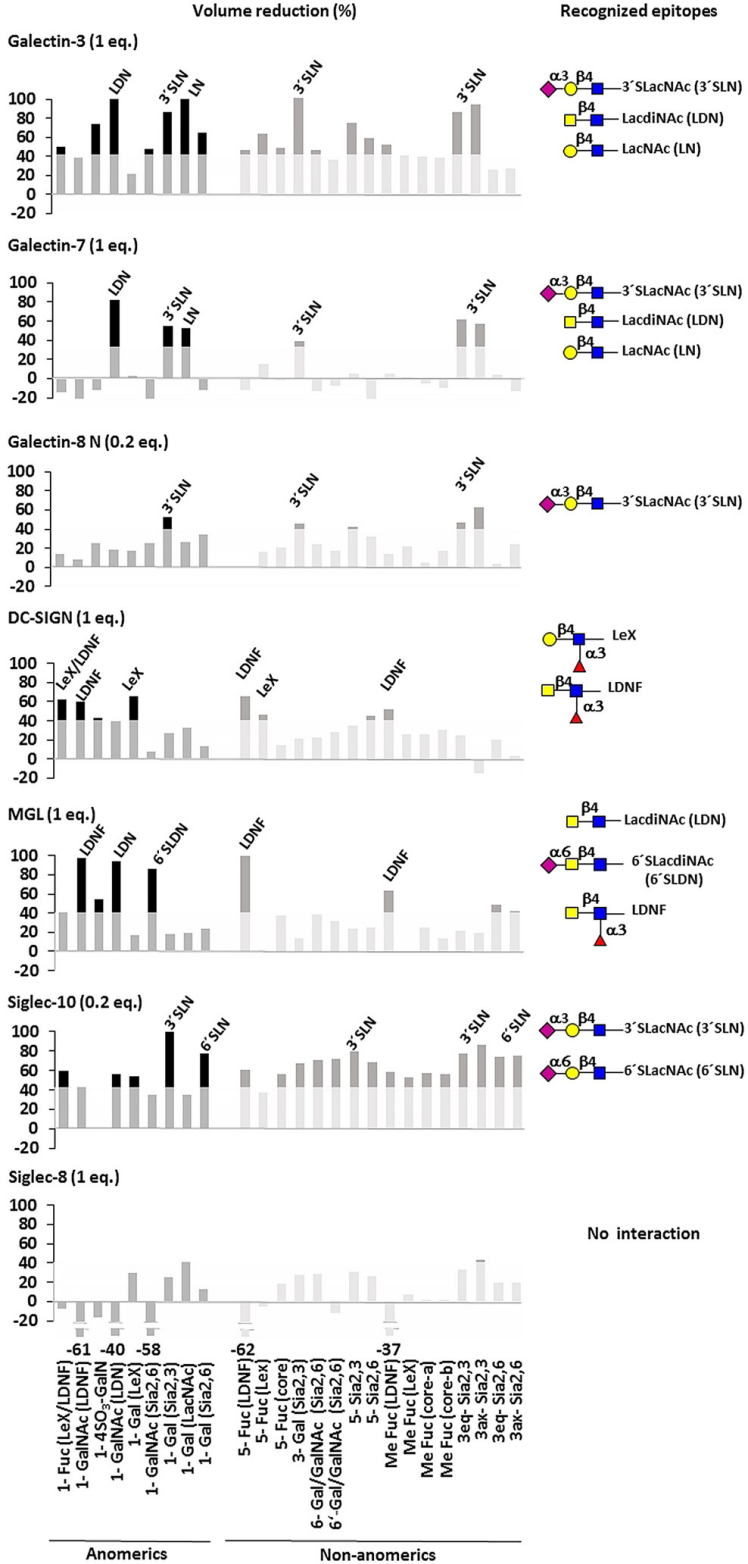
Graphical bar representation for the % of cross peak volume reduction on ^1^H,^13^C‐HSQC of RBD upon adding the different lectins. From top to bottom: galectin‐3 (1 equiv), galectin‐7 (1 equiv), galectin‐8N (0.2 equiv), DC‐SIGN (1 equiv), MGL (1 equiv), Siglec‐10 (0.2 equiv), Siglec‐8 (1 equiv). Each cross peak is identified with a number corresponding to the position on the pyranose, the residue, and the epitope. An arbitrary threshold (transparent square) is used to highlight the most affected signals. SNFG representation of the major deduced epitopes on the RBD are on the right.

**Figure 5:**
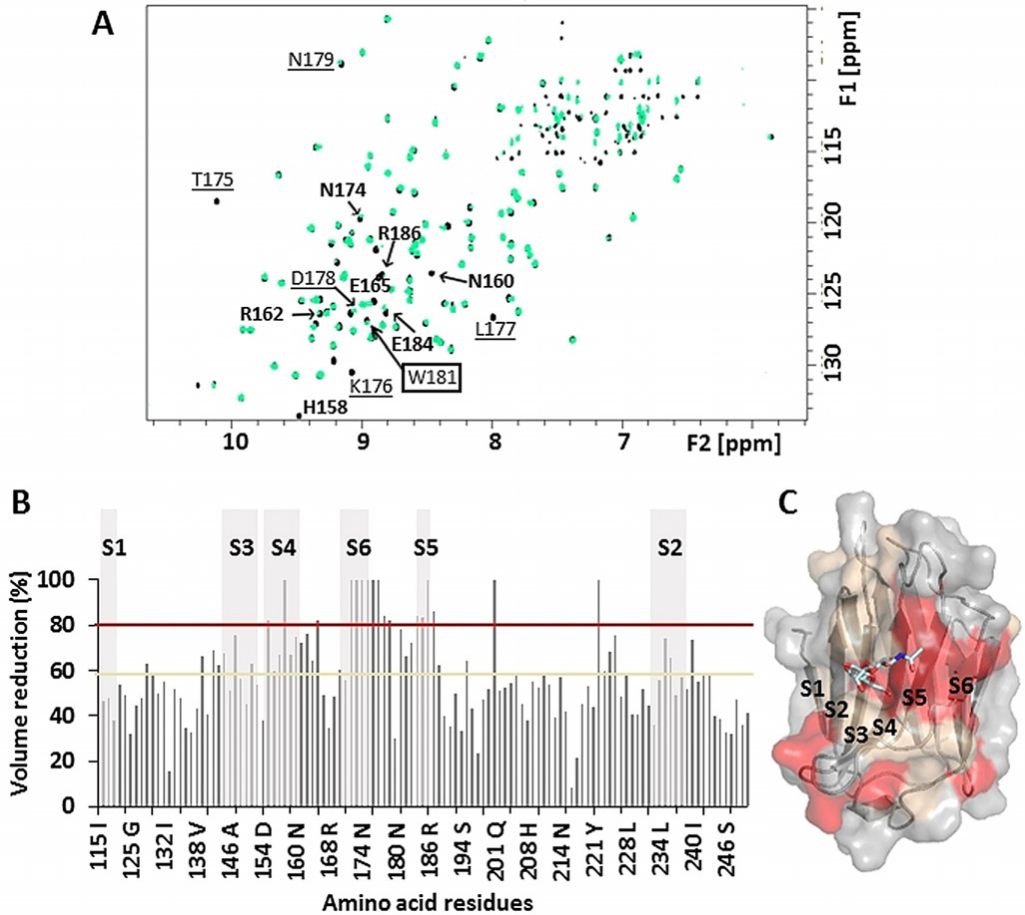
Galectin‐3/RBD interaction deduced by NMR from the lectin perspective. A) Superimposition of the ^1^H,^15^N‐TROSY for free galectin‐3 (black) and galectin‐3/RBD (green). Some affected cross peaks are annotated: amino acids involved in LacNAc interactions are bolded; those interacting with α2‐3 sialic acid are underlined. The key signal of W181 is squared. B) % cross peak volume reduction on the ^1^H,^15^N‐TROSY upon addition of galectin‐3. S1–S6 β‐strands are depicted with grey boxes. Red and wheat horizontal lines are baselines for cross peaks suffering 80–100 % reduction and 60–80 % reduction, respectively. C) Cartoon and surface representation of galectin‐3 bound to LacNAc (PDB 1A3K) according to the X‐Ray structure. Amino acids are coloured based on their perturbation (% volume reduction) due to RBD binding (threshold in B).

**Figure 6:**
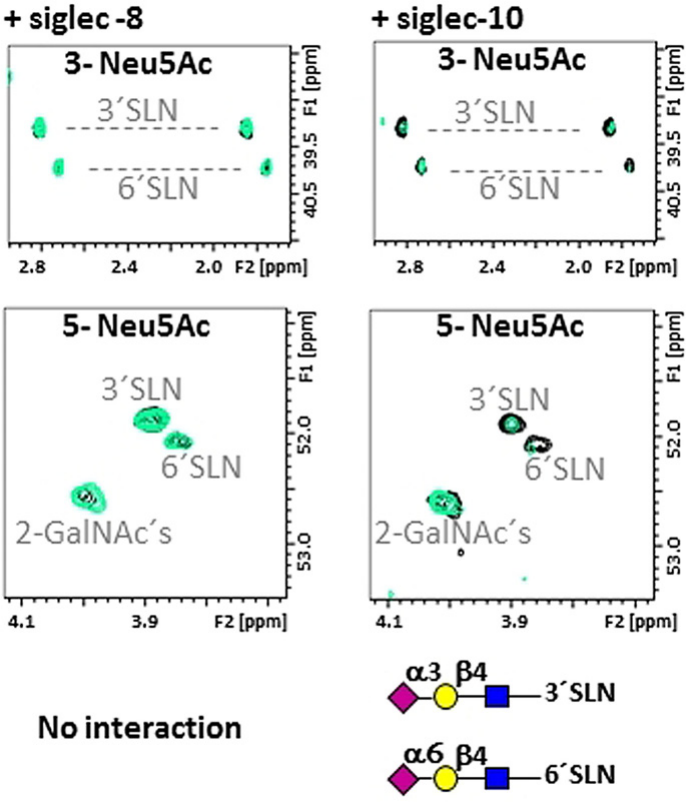
The interaction of RBD with Siglecs ‐8 and ‐10 from the glycan perspective. Different regions of the ^1^H,^13^C‐HSQC of RBD alone (in black) and with 1 equiv. of Siglec‐8 (left, superimposed in green), and with 0.2 equivalents of Siglec‐10 (right, superimposed in green). Top and middle: regions for the C3‐H3 and C5‐H5 correlations of Neu5Ac. In the presence of Siglec‐8, no signal is affected, indicating that there is no interaction, while in the presence of Siglec‐10, the signals of Neu5Ac, both α2‐3 and α2‐6 linked are affected, indicating that Siglec‐10 interacts with the RBD through these epitopes. The graphical bar representation for % of volume reduction of cross peaks on the ^1^H,^13^C‐HSQC of RBD upon adding Siglec‐8 and ‐10 are in Figure 4.

**Figure 7:**
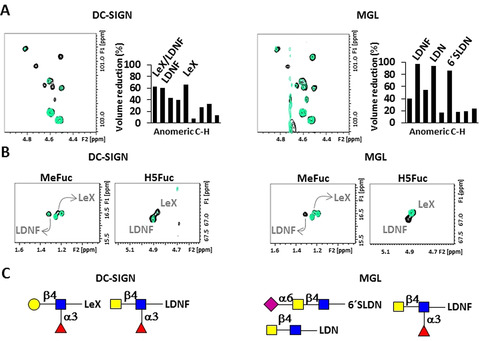
Different interactions of the RBD with C‐type lectins DC‐SIGN and MGL from the glycan perspective. Selected regions of the ^1^H,^13^C‐HSQC of RBD alone (in black), with 1 equiv. of DC‐SIGN (left, superimposed in green), and with 0.2 equivalents of MGL (right, superimposed in green). A) Anomeric region and graphical bar representation for the % of volume reduction (also in Figure 4 with additional cross peaks) B) Specific regions for C6‐H6 (Me) and C5‐H5 correlations of Fuc in LDNF and LeX. C) Epitopes on RBD recognised by both lectins, as SNFG symbols.

It has been recently proposed that the spike glycoprotein is specifically recognized by C‐type lectins (Dendritic Cell‐Specific Intercellular adhesion molecule‐3‐Grabbing Non‐integrin (DC‐SIGN) and Macrophage Galactose‐type lectin (MGL)) and by Siglecs expressed in the lung microbiota.^[12]^ Thus, we focused on deducing the specific epitopes responsible for these interactions. Moreover, we also studied the interaction with several human galectins (galectins‐3, 7 and 8), which are involved in inflammation.[^49^, ^50^] It has been proposed that galectin inhibitors may modulate the cytokine storm associated to COVID‐19 as well as interfering with viral attack.^[51]^ Thus, NMR experiments both from the glycan and from the lectin perspectives were carried out to monitor the lectin/RBD interactions. From the glycan point of view, the cross peak intensities of the ^13^C‐labelled glycans described above were compared to those recorded in the presence of the lectins (Figures 3, 4, 6, and 7). Alternatively, ^1^H,^15^N‐TROSY/HSQC experiments allowed analysing the line width perturbations of the amide NMR signals of the ^15^N‐labelled lectins upon RBD addition (Figure 5 and Figures S4–7). Although signal broadening depends on many factors and cannot be directly related with binding affinity, it allowed us to discern the glycan specificities among the lectins studied.

### The Interaction with Galectins

Upon addition of galectin‐3, the ^1^H,^13^C‐HSQC spectrum of the RBD glycans showed significant, but selective, reductions in the intensity of diverse cross peaks (Figures 3 and 4). Intensity attenuation was more pronounced for peaks from LDN, LacNAc and 3′SLacNAc. The observed dramatic signal broadening evidence the presence of dynamic processes in the intermediate exchange regime in the NMR chemical shift time scale and thus, the presence of a significant interaction.

Additional information was obtained by observing the changes in the lectin ^1^H,^15^N cross peaks (Figure 5). The results strongly suggest that the interaction with the RBD affects the canonical LacNAc binding site of galectin‐3.^[23]^ In fact, the ^1^H,^15^N‐TROSY spectrum of the galectin‐3/RBD sample (Figure 5) exhibited the complete disappearance of specific cross peaks, such as that for H158, conserved among galectins and participating in hydrogen bonding with 4OH‐Gal, or R186, involved in hydrogen bonding with the GlcNAc moiety in the complexes with LacNAc. W181, conserved and key for the CH‐π stacking interaction with Gal, was also disturbed. Additionally, the whole S5 or S6 β‐strand (V172‐L177) along the loop 177–180 were highly affected. In particular, the T175‐N179 region is hardly modified by LacNAc, but greatly perturbed when LacNAc is α2‐3 sialylated (Figure S3). Thus, these data confirm that galectin‐3 binds the RBD through the canonical Gal binding site by specifically recognising terminal LN, LDN and 3′SLN epitopes on the RBD.^[23]^

We next studied the interaction of the RBD with the N‐terminal domain of galectin‐8 (galectin‐8N), a tandem repeat lectin whose N‐terminal domain has partially overlapping glycan binding preferences with galectin‐3,[^52^, ^53^] although with diverse affinities for the same epitopes.[^54^, ^55^] Strikingly, when the RBD and galectin‐8N were mixed in a 1:1 ratio, the same conditions for galectin‐3, the sample became cloudy and unmanageable for NMR experiments. Thus, a 1:0.2 ratio (^13^C‐RBD:galectin) was used, resulting in a clear sample that allowed recording the ^1^H,^13^C‐HSQC (Figure 4). The observed cross peak signal reduction was now more selective than for galectin‐3, and showed that galectin‐8N binds mainly to the 3′SLN RBD glycan epitope. The interaction from the lectin perspective (Figure S4) showed that the most affected residues on galectin‐8N were around the canonical glycan binding site. These results permit not only underline the different glycan binding preferences between both galectins towards the RBD in terms of epitopes, but also the different recognition phenomena that take place when the binding epitopes are differently exposed or hidden, especially in multivalent presentations, as also highlighted by others.^[52]^

Finally, the prototype galectin‐7 was tested. Galectin‐7 contains two identical glycan binding sites, forms non‐covalent homodimers, and displays the lowest glycan affinities reported among galectins.[^52^, ^56^] The NMR analysis of the RBD glycans upon addition of 1 equivalent of galectin‐7 revealed that the most perturbed signals correspond to the LDN epitope, with those for LN and 3′SLN also affected to a lesser extent (Figure 4). These results agree with reported data that showed that acetylation of terminal Gal moieties increased the affinity for galectin‐7,^[21]^ although contrast respect to 3′SLN which was reported to bind weaker than LN.[^21^, ^57^] From the lectin perspective, the cross peak intensity loss upon RBD addition affected not only amino acids at the lactose binding pocket (W70, H50, V60, V61, N63, N52, E74), but also far from this site (T57, S58, Q67, loops), at the F face (G76, R113, Y106), and even at the dimer interface (V89, L90), reflecting that most of the protein is actually affected by the interaction (Figure S5). This evidence agrees with previous studies that claimed an inter‐domain communication upon lactose binding.^[58]^

### The Interaction with Siglecs

The combination of the RBD with Siglec‐8 produced no changes neither on the ^1^H,^13^C‐HSQC of the ^13^C‐glycans at the RBD (Figure 6 and Figure S6), nor in the ^1^H,^15^N‐TROSY of ^15^N‐Siglec‐8 (Figure S6). Thus, Siglec‐8 does not recognize any glycan on the RBD, in agreement with the tight glycan binding selectivity of this lectin that binds terminal 3′SLN and SLeX, only when they are sulfated at Gal 6.[^22^, ^59^] This chemical modification is not present in our glycosylated RBD since it would be readily identified due to the characteristic chemical shifts of a sulfated C6‐Gal. A completely different situation was found for Siglec‐10, for which the addition of 0.2 equivalents of lectin to the RBD caused a general reduction of the cross‐peak intensities of the ^13^C‐labelled glycans. The effect was more pronounced for the signals of terminal 3′SLN and 6′SLN epitopes (Figure 6), in agreement with the reported selectivity for this lectin.^[60]^ Although a preference for 6′SLN over 3′SLN has been described, this is not appreciable from the NMR data. Information from the lectin perspective was not possible in this case due to the lack of access to a suitable ^15^N‐labelled lectin for NMR.

### The Interaction with C‐Type Lectins DC‐SIGN and MGL

The presence of 1 equivalent of DC‐SIGN caused a selective intensity decrease on specific glycan ^1^H,^13^C‐HSQC cross peaks of the RBD (Figure 7). In terms of terminal epitopes, the LeX and LDNF signals were the most affected (Figures 4 and 7).

This fact is in agreement with the reported preference of DC‐SIGN for these moieties, in which key interactions are provided by the Fuc residue that binds at the calcium binding site of the lectin.[^20^, ^61^] The comparison with the effects produced by 1 equivalent of MGL were markedly different (Figures 4 and 7), permitting to identify the diverse binding preferences of both lectins. Indeed, the presence of MGL produced an exquisite selective reduction of the signals corresponding to GalNAc‐containing epitopes, with the exception of 4SulLDN (Figures 4 and 7). Thus, terminal LDN, its α2‐6 sialylated version (6′SLDN), and the fucosylated LDN (LDNF) are the glycans specifically recognized by MGL.

With respect to the lectin binding site, the cross peaks on the ^1^H,^15^N‐TROSY of DC‐SIGN (Figure S7) exhibited differential intensity loss upon addition of RBD. The most affected residues belong to the calcium binding site, directly involved in interactions with the bound Fuc (N365, D366, N367, K368). Additionally, the signals for F313 and F374 were completely absent in the presence of the RBD, confirming the placement of Gal/GalNAc close to F313.[^20^, ^61^, ^62^] Interestingly, a number of residues at a secondary calcium site (D320, L321, Q323, G325, T326 and W327) were also affected. The results for MGL were completely different, reflecting the different dynamic properties of both lectins. The presence of 0.5 equivalents of RBD produced the homogeneous intensity reduction for most of the lectin with the exception of the C‐terminal fragment, while 1 equivalent produced the complete disappearance of all the NMR signals in the ^1^H,^15^N‐HSQC (Figure S8). In order to confirm that the MGL glycan binding site was indeed involved, competition experiments with a simple GalNAc sugar were performed (Figure S9). Suitably, the addition of 1 equivalent of GalNAc produced the recovery of the NMR signals of the lectin, confirming that the RBD and GalNAc compete for the same binding site.

**Figure 8:**
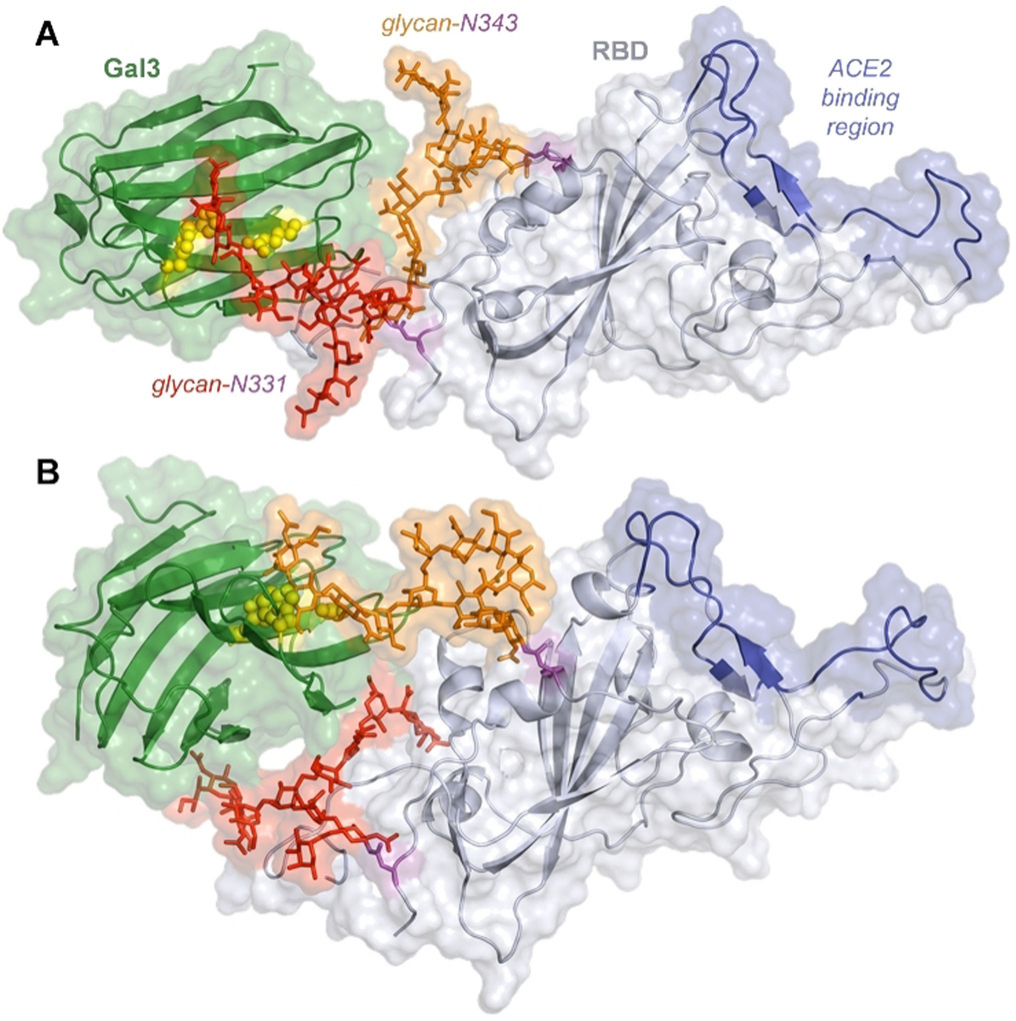
3D models for 1:1 complexes of galectin‐3 (PDB 4R9A) with the 3′SLacNAc epitope attached at both RBD glycosylation sites (PDB 6VSB), according to selected snapshots of 1 μs MD simulations. Binding through glycans at N331 (A) and N343 (B). Galectin‐3 and RBD are shown as green and grey cartoons, respectively. The ACE2 binding region of RBD is in blue. Glycans at N331 and N343 are in red and orange sticks, respectively. Glycosylated Asn are in magenta. Key binding residues H162, R62, W181 are shown as yellow spheres.

Once the interacting glycan epitopes were experimentally assessed, putative 3D structures were generated for the complexes formed between the glycosylated RBD and several lectins (galectin‐3, galectin‐7, galectin‐8N and DC‐SIGN) using the coordinates of the X‐ray crystal structures of the lectins (PDB 4R9A, 4GAL, 5GZF, 1SL5, respectively) and that of the RBD within the full S glycoprotein structure (PDB 6VSB) as described in the supporting information. For MGL, an homology model was built since no crystallographic structures are available. Given the existence of two glycosylation sites at the RBD, two 1:1 complexes were generated for each RBD/lectin system, one for the glycan at N331 and a second for that at N343. Molecular dynamics simulations (1 μs) were run for each complex to produce fully equilibrated structures in water solution. The simulations revealed a multitude of highly dynamical glycan‐receptor contacts in addition to those established at the canonical sugar binding sites, in agreement with the NMR observations. The formation of fleeting unspecific interactions between RBD glycans and the receptor can be appreciated, often creating an interface between the two proteins. As an example, a 3D perspective of the possible interaction of the glycosylated RBD to galectin‐3 is shown in Figure 8, while those for the other lectins are gathered in the SI. Interestingly, interaction with galectin‐3 takes place on the opposite side of RBD with respect to the ACE2 recognition region. As a general trend, complexes involving glycans at N343 are more compact and display larger intermolecular contacts than those involving the solvent‐exposed glycans at N331 (see SI).

Overall, our study has allowed identifying N‐linked glycan epitopes located in the RBD of the spike protein from SAR‐CoV‐2 that serve for the recognition of host lectins, which may contribute to viral infection and subsequent immune exacerbation. An additional analysis of binding and competition using the fully glycosylated spike trimer, will help us test the possible interfering ability of these human lectins either by ACE2 binding competition or by hampering the fusion of the virus with human cells.

## Conclusion

The RBD fragment of the SARS‐CoV‐2 S glycoprotein with ^13^C glycan labelling has been generated. The great sensitivity provided by ^13^C opens the door to significant opportunities for exhaustive NMR analysis of its glycoprofile and its molecular recognition features. Thus, by employing an NMR‐based methodology, which avoids sample digestions and derivatizations, most of the ^1^H and ^13^C NMR glycan resonances of the intact (folded) glycoprotein in solution have been assigned, allowing to characterize the specific terminal glycan epitopes exposed on the antennae of the RBD N‐glycans. Although the current analysis do not allow for fully quantitative occupancy determination and site specific identification at N331 and N343, it has provided unprecedented structural details. Thus, besides the expected LN, 3′SLN, and 6′SLN terminal moieties, the presence of LDN and its fucosylated LDNF derivative have been assessed. Whereas the former has been detected in a trimer‐stabilized version of the SARS‐CoV‐2 S protein, the presence of the LDNF epitope was unexpected. Indeed,^[15]^ LDN motifs have been found on several mammalian glycoproteins and observed in HEK293‐produced glycoproteins.^[63]^ In contrast, the LDNF epitope has been mainly related to pathogens. Additionally, 4‐O‐sulfated and α2‐6 sialylated LDN derivatives, not previously reported either, have also been identified as terminal epitopes, together with the LeX epitope. Overall, our analysis highlights the presence of important levels of N‐acetyl‐galactosylation and hyper‐fucosylation at the terminal chains of the RBD N‐glycans, revealing glyco‐epitopes not observed in previous MS‐based analysis.[^13^, ^14^, ^15^, ^16^] Interestingly, the comparison with a different glycoprotein produced exactly under the same conditions suggests a relationship between the observed high levels of GalNAc and Fuc contents with the protein structure. The exhaustive NMR analysis has also allowed disclosing the main N‐glycan scaffold, being complex biantennary, core fucosylated, while lacking bisecting GlcNAc and elongated antennas involving type I polyLacNAc sequences.

The interaction of the glycosylated RBD with a panel of human lectins has also been scrutinized. The ^13^C‐glycan labeling of the RBD has permitted to exploit the ^1^H,^13^C‐HSQC spectrum of the RBD to report on the specific glycan epitopes recognized by each lectin, affording the corresponding glycan binding selectivity. Thus, while galectins‐3 and ‐7 recognize the LN, LDN and 3′SLN motifs on the RBD, galectin‐8N seems to prefer exclusively the 3′SLN epitope. Siglecs‐8 and ‐10 demonstrated markedly differences, with Siglec‐8 unable to recognize any of the glycan epitopes on the RBD, while Siglec‐10 interacting with both 3′SLN and 6′SLN. For the C‐type lectins, DC‐SIGN exhibited selectivity for the two fucosylated terminal epitopes LeX and LDNF, while MGL showed exquisite selectivity for all GalNAc containing epitopes, except for the 4‐O‐sulfated derivative. The complementary information obtained from the ^15^N‐lectin based experiments permitted to assess that binding occurs through the canonical glycan binding site for each of the lectins (except for Siglec‐10). Importantly, all the binding studies have been carried out by using the intact (folded) form of the RBD glycoprotein in solution, allowing to propose atomistic 3D models for the corresponding complexes.

This study paves the way to unveiling the interlaces roles of glycosylation patterns and cell receptors in SARS‐CoV‐2 infection mechanisms in the cell, particularly the recognition of tissue‐dependent ACE2 by full‐length glycosylated spike protein (S). Such studies are currently ongoing in our labs.

## Conflict of interest

The authors declare no conflict of interest.

## Supporting information

As a service to our authors and readers, this journal provides supporting information supplied by the authors. Such materials are peer reviewed and may be re‐organized for online delivery, but are not copy‐edited or typeset. Technical support issues arising from supporting information (other than missing files) should be addressed to the authors.

Supplementary
