# Supplementary material for: Structural Characterization of N‐Linked Glycans in the Receptor Binding Domain of the SARS‐CoV‐2 Spike Protein and their Interactions with Human Lectins
Source: Angew Chem Int Ed Engl. 2020 Oct 22;59(52):23763–71. doi: 10.1002/anie.202011015 (PMC7894318; doi:10.1002/anie.202011015)
Supplement: Supplementary file 1 — Supplementary [file ANIE-59-23763-s001.pdf]

Angewandte Chemie
Eine Zeitschrift der Gesellschaft Deutscher Chemiker

Supporting Information

Structural Characterization of N-Linked Glycans in the Receptor Binding Domain of the SARS-CoV-2 Spike Protein and their Interactions with Human Lectins

Maria Pia Lenza, Iker Oyenarte, Tammo Diercks, Jon Imanol Quintana, Ana Gimeno, Helena Coelho, Ana Diniz, Francesca Peccati, Sandra Delgado, Alexandre Bosch, Mikel Valle, Oscar Millet, Nicola G. A. Abrescia, Asís Palazón, Filipa Marcelo, Gonzalo Jiménez-Osés, Jesús Jiménez-Barbero, Ana Ardá,* and June Ereño-Orbea**

anie_202011015_sm_miscellaneous_information.pdf

Author Contributions

M.L. Data curation: Supporting; Formal analysis: Supporting; Investigation: Lead; Methodology: Equal; Writing—Review & Editing: Supporting
I.O. Formal analysis: Supporting; Investigation: Equal; Methodology: Equal
T.D. Investigation: Equal; Methodology: Equal; Resources: Supporting; Writing—Original Draft: Supporting; Writing—Review & Editing: Supporting
J.Q. Data curation: Supporting; Formal analysis: Supporting; Investigation: Supporting; Methodology: Supporting; Validation: Supporting; Writing—Review & Editing: Supporting
A.G. Formal analysis: Supporting; Investigation: Supporting; Methodology: Supporting; Software: Supporting; Validation: Supporting; Writing—Review & Editing: Supporting
A.B. Data curation: Supporting; Investigation: Supporting; Methodology: Supporting; Validation: Supporting
H.C. Formal analysis: Supporting; Investigation: Supporting; Methodology: Supporting; Validation: Supporting
A.D. Formal analysis: Supporting; Investigation: Supporting; Methodology: Supporting; Validation: Supporting
F.P. Formal analysis: Supporting; Investigation: Supporting; Methodology: Supporting; Software: Supporting; Validation: Supporting; Writing—Review & Editing: Supporting
S.D. Formal analysis: Supporting; Investigation: Supporting; Methodology: Supporting; Validation: Supporting
M.V. Conceptualization: Supporting; Funding acquisition: Supporting; Investigation: Supporting; Validation: Supporting; Writing—Review & Editing: Supporting
O.M. Conceptualization: Supporting; Formal analysis: Supporting; Funding acquisition: Supporting; Investigation: Supporting; Validation: Supporting; Writing—Review & Editing: Supporting
N.A. Conceptualization: Supporting; Formal analysis: Supporting; Funding acquisition: Supporting; Investigation: Supporting; Validation: Supporting; Writing—Review & Editing: Supporting
A.P. Conceptualization: Supporting; Funding acquisition: Supporting; Investigation: Supporting; Supervision: Supporting; Validation: Supporting; Writing—Review & Editing: Supporting
F.M. Data curation: Supporting; Formal analysis: Supporting; Investigation: Supporting; Methodology: Supporting; Supervision: Supporting; Validation: Supporting; Writing—Original Draft: Supporting
G.J. Conceptualization: Supporting; Funding acquisition: Supporting; Investigation: Supporting; Methodology: Supporting; Software: Supporting; Supervision: Supporting; Validation: Supporting; Writing—Review & Editing: Supporting
A.A. Conceptualization: Equal; Formal analysis: Equal; Investigation: Equal; Methodology: Equal; Supervision: Equal; Validation: Equal; Writing—Original Draft: Equal; Writing—Review & Editing: Equal
J.E. Conceptualization: Equal; Data curation: Equal; Formal analysis: Equal; Funding acquisition: Equal; Investigation: Equal; Methodology: Equal; Supervision: Equal; Validation: Equal; Writing—Original Draft: Equal; Writing—Review & Editing: Equal.

SUPPORTING INFORMATION

TABLE OF CONTENTS

1.	Materials	S3
2.	NMR experiments	S4
3.	Molecular Modeling	S5
4.	Figure S1	S7
5.	Table S1	S8
6.	Figure S2	S9
7.	Figure S3	S9
8.	Figure S4	S10
9.	Figure S5	S11
10.	Figure S6	S12
11.	Figure S7	S13
12.	Figure S8	S14
13.	Figure S9	S14
14.	Figure S10	S15
15.	Table S2	S15
16.	Figure S11	S15
17.	Figure S12	S16
18.	Figure S13	S16
19.	Figure S14	S17
20.	Table S3	S17
21.	Bibliography	S18

1. Materials

Construct design, expression and purification of the glycosylated proteins RBD and FcεRIα. The DNA codifying the S^B domain of the RBD (amino acid residues 328-533) from the spike protein of SARS-CoV-2 (UniprotKB P0DTC2) was codon optimized for expression in human cells and synthesized by Genscript Biotech. The construct was subcloned into pHLsec vector[1] between AgeI and KpnI restriction sites, such that a 6x His tag was added to the C-terminus of RBD. RBD was transiently transfected into HEK239F (Thermo Fisher Scientific) suspension cells with the transfection reagent FectoPRO (Polyplus Transfections). Labelling of the N-glycans on RBD was achieved by adding 0.6 g of ^{13}C D-glucose (Cambridge Isotope Laboratories, Inc.) to 200 mL of transfected HEK293F cells.[2,3] Transfected cells were incubated at 37 °C, 180 rpm, 8% CO_2 in a Minitron Pro shaker (Infors HT) for 6–7 days. Cells were harvested by centrifugation at 6371 xg for 20 min, and supernatants were retained and filtered using a 0.22 µm Polysterene sterile filters (Corning). Supernatants were passed through a HisTrap Ni-NTA column (GE Healthcare). Eluted fractions containing RBD were pooled, concentrated, and separated on a Superdex 75 Increase 10/300 size exclusion column (GE Healthcare).

FcεRIα[4] was expressed in HEK239F (Thermo Fisher Scientific) suspension cells as described for the RBD. Protein was purified through a HisTrap Ni-NTA column (GE Healthcare) followed by size exclusion chromatography on a Superdex 200 Increase 10/300 column (GE Healthcare).

Construct design, expression and purification of human lectins. The carbohydrate recognition domains (CRD) of siglec-8 (residues 17-155), MGL (residues 181-316), galectin-3 (residues 114-250), the full length galectin-7 (residues 1-136) and the N-terminal domain of galectin-8 (galectin-8N, residues 1-155) were cloned into pET-43.1a (+) (Addgene), pET-21a (+) (NZYTech), pET-21a (+) (Addgene), pET-22b (+) (Novagene) and pET-21b (+) (Addgene) vectors, respectively. Expression and purification of unlabelled and ^{15}N-labelled protein was performed as described elsewhere.[5–7]

The DNA codifying the full-length extracellular domain of siglec-10 (amino acid residues 17-537) (UniprotKB Q96LC7) fused to mVENUS[8] was synthesized, codon optimized for expression in human cells and cloned into pHLsec vector[1] between AgeI and KpnI restriction sites by Genscript Biotech. The transient expression of siglec-10 in HEK293F suspension cells was achieved as described for RBD. Pure protein was obtained by HisTrap Ni-NTA column (GE Healthcare) followed by Superdex 200 Increase 10/300 size exclusion chromatography (GE Healthcare).

2. NMR experiments

General information. All proteins, except DC-SIGN and MGL, were used in phosphate saline (PBS) buffer pH 7.4, in either H_2O (with 10% D_2O added) or pure D_2O. Buffer for DC-SIGN was 25 mM Tris pH 8, 150 mM NaCl and 5 mM $CaCl_2$; and for MGL was 10 mM Tris pH 7.5, 75 mM NaCl, 20 mM $CaCl_2$, in either H_2O (with 10% D_2O added) or pure D_2O.The total volume of the NMR samples was 300 µL that were filled into Shigemi NMR tubes with 5 mm outer diameter. The buffer pH was measured with a Crison Basic 20 (Crison Instruments SA, Barcelona, Spain) pH meter and adjusted with the required amount of NaOH and HCl, or NaOD and DCl. All spectra were measured on an 800 MHz BRUKER AVANCE III spectrometer, equipped with a TCI cryo-probe with z-gradient coil, with exception of the experiment using ^{15}N-labelled MGL CRD that were carried out on an 600 MHz BRUKER AVANCE III spectrometer, equipped with a 5mm inverse detection triple-resonance z-gradient cryogenic probe (CP-TCI), using TopSpin 3.2.7 (BRUKER) for both data acquisition and processing.

^{1}H-^{13}C Signal assignment experiments for RBD glycoprotein. RBD with ^{13}C-labelled-glycans was concentrated to 625 µM in buffered (PBS) D_2O, and the following suite of spectra were recorded at 310 K for glycan signal assignment: *i*) A high-resolution 2D H,^{13}C HSQC with States-TPPI sampled constant t_1(^{13}C) time evolution during 22 ms and 47 Hz F1(^{13}C) digital resolution, 3.7 ms $^{1}J_{CH}$ evolution and d1 = 1s total interscan delay; this spectrum encodes signals with an odd or even number of ^{13}C neighbours (excluding the decoupled ^{13}CO groups) with a negative or positive sign, respectively. *ii*) Two 3D [H']C',CH encoded C'C-TOCSY experiments[9,10] with semi-constant t_1(^{13}C') and t_2(^{13}C) evolution, gradient echo/antiecho coherence selection in F2(^{13}C), 3.125 ms $^{1}J_{CH}$ evolution and d1 = 1.26s total interscan delays; the C'C-TOCSY was implemented using FLOPSY16 (with a B_{RF} = 14.3 kHz centered at 60 ppm) for 12.25 or 26.4 ms mixing time, where the latter yielded almost complete correlation for all saccharide moieties. The digital resolution was 143 Hz(F1) x 189 Hz(F2), reached with a non-uniform sampling (NUS) level of 17%. *iii*) A complementary 3D H'[C'],CH encoded C'C-TOCSY[11] was recorded similarly, using 26.4 ms FLOPSY16 mixing, semi-constant t_1(H') sampling (States-TPPI) up to 21.5 Hz digital resolution, and 16% NUS. *iv*) Two 3D H',CH NOESY-HSQC experiments with 3.125 ms $^{1}J_{CH}$ evolution and d1 = 1.09s total interscan delays, and 200 ms or 300 ms NOE evolution time. The digital resolution was 25 Hz(F1: H') x 189 Hz(F2:13C), reached with a non-uniform sampling (NUS) level of 17%. All cited experiments implemented residual HDO signal presaturation during d1 at 5 µW, and ^{13}C decoupling during the 55ms ^{1}H FID acquisition time (resulting in 16.8 Hz digital resolution) using GARP4 with a B_{RF} of 4.5 kHz.

Molecular interaction studies by lectin detected $^1H,^{15}N$-TROSY. 2D $^1H,^{15}N$ BEST-TROSY spectra of [U-^{15}N] labelled human lectins were recorded using 60 μM concentration of the lectin, and adding μL volumes of unlabelled RBD stock solutions in the same buffer, to achieve 1:1, 1:0.4 or 1:0.5 ratios. The lectin backbone resonance assignment was done according to database (BMRB) deposited data (bmrb 4909 for galectin-3,[12] bmrb 17826 for galectin-7,[13] bmrb 27854 for DC-SIGN,[14] and bmrb 25798 for Siglec-8 [15]. Backbone resonance assignment for MGL and galectin-8N was carried out by us following standard protocols.[7] In the case of MGL a $^1H,^{15}N$-HSQC spectra using 180 μM concentration of the lectin, and adding μL volumes of unlabelled RBD stock solution in the same buffer, to achieve 1:0.5 and 1:1 ratios, were recorded. A suplementary $^1H,^{15}N$-HSQC experiment was carried out after addition of 1 equivalent of α-methyl GalNAc. Data analysis, including cross peak volume, was carried out in CcpNmr Analysis V2 software.[16]

Molecular interaction studies by RBD glycan detected $^1H,^{13}C$-HSQC. 2D $^1H,^{13}C$-HSQC spectra of RBD with ^{13}C-glycan labelling were recorded using 60 μM of RBD, and adding μL volumes of the corresponding unlabelled lectin in the same buffer, to achieve 1:1 or 1:0.2 ratios. Cross peak volume integration was carried out in Bruker TopSpin3.5pl6 software.

3. Molecular modelling

Molecular modelling. The initial structures for the lectins/RBD complexes were built using the X-ray crystal structures of the carbohydrate recognition domains (CRD) of human galectin-3 (PDB ID 4R9A),[17] galectin-7 (PDB ID 4GAL),[18] galectin-8 (N-terminal domain, PDB ID 5GZF)[19] and DC-SIGN (PDB ID 1SL5).[20] For MGL, and homology model was generated using the related asialoglycoprotein receptor (ASGPR, PDB ID 5JPV)[21] as a template with the high-resolution comparative modelling module of Rosetta software.[22] In parallel, fully glycosylated models of SARS-CoV-2 RBD were built based on the cryo-EM structure of the spike protein on the prefusion conformation (PDB ID 6VSB),[23] which was later curated[25] and available at the CHARMM-GUI online server.[26] A non-glycosylated initial geometry for RBD comprising residues R328 to L533 was extracted from this model, and a polyhistidine tag (KHHHHHH) was added at C-terminus using PyMol software[27] to resemble the experimental conditions as closely as possible. Residues N331 and N343, both located near the N-terminal region, were then N-glycosylated using the *glycoprotein builder* available at the GLYCAM-Web online server.[28] The glycans modelled for each receptor/RBD complex were those showing the highest affinity by NMR spectroscopy and shared the same biantennary core (see Supporting Information). For simplicity, identical glycans with equal antennae were modelled

at both N-glycosylation sites for each receptor/RBD complex, producing a total of ten starting models. In each case, the α3 branch of the RBD glycans were docked into the binding sites of the receptors by fitting the coordinates of the modelled carbohydrates mostly responsible for binding (i.e. D-Gal for galectin-3 and galectin-8; D-GalNAc for galectin-7 and MGL; L-Fuc for DC-SIGN) with those of the same or related ligands found in the X-ray structures of the lectins in the bound state. Finally, molecular dynamics simulations for each binary RBD-receptor complex were run with the AMBER suite,[29,30] using the ff14SB[31] and GLYCAM06[32] force fields. Initial structures were neutralized with either Na^+ or $Cl^–$ ions and set at the centre of a cubic TIP3P water[33] box with a buffering distance between solute and box of 10 Å. For each complex, we followed a two-stage geometry optimization approach: the first stage minimizes only the positions of solvent molecules and ions, and the second stage is an unrestrained minimization of all the atoms in the simulation cell. The systems were then heated by incrementing the temperature from 0 to 300 K under a constant pressure of 1 atm and periodic boundary conditions. Harmonic restraints of 10 kcal mol^{-1} were applied to the solute, under the Andersen temperature coupling scheme.[34,35] The time step was kept at 1 fs during the heating stages, allowing potential inhomogeneities to self-adjust. Water molecules were treated with the SHAKE algorithm[36] such that the angle between the hydrogen atoms is kept fixed through the simulations. Long-range electrostatic effects were modelled using the particle mesh Ewald method.[37] An 8 Å cutoff was applied to Lennard-Jones interactions. Each system was equilibrated for 2 ns with a 2 fs time step at a constant volume and temperature of 300 K. Production was run as a 200 ns NVT trajectory at 300 K with a time step of 2 fs using the Andersen thermostat. Representative snapshots at the end of the production trajectories were obtained using the *cpptraj* module of AMBER and rendered with PyMol.

4. Figure S1

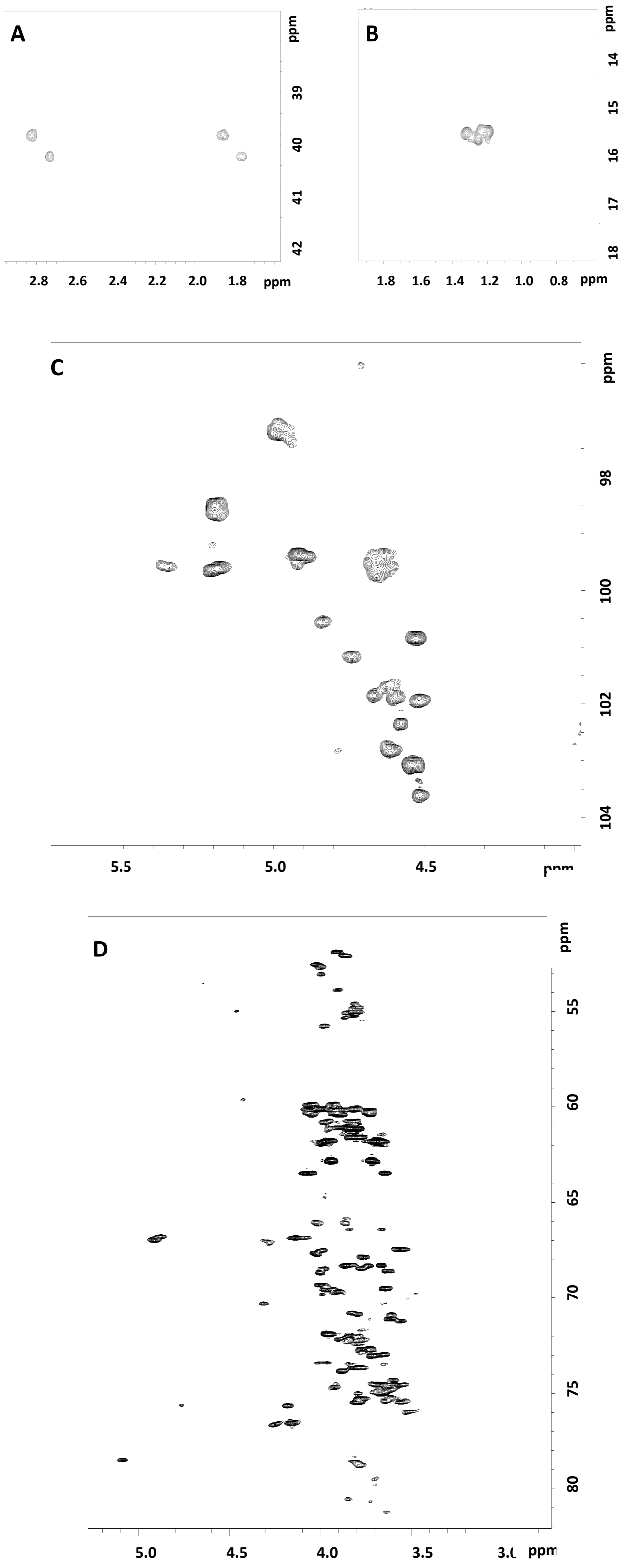

Figure S1. Different regions of the $^{1}H,^{13}C$-HSQC spectrum of RBD with ^{13}C-glycans at 310K. A. B C6,H6 (Me) correlations of the Fuc residues. C. Anomeric region. D. Region for most pyranose C,H correlations.

5. Table S1

Table S1. ^{1}H and ^{13}C chemical shifts for RBD N-glycans at 310K. Each column correspond to a residue identified on top in bold in the corresponding epitope. nd: not determined.

	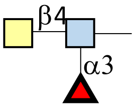 **Fuc (LDNF)**	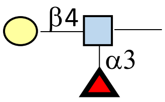 **Fuc (Le^{x})**	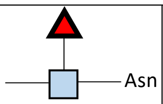 **Fuc (core) (a,major)**	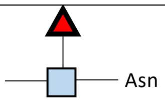 **Fuc (core) (b,minor)**	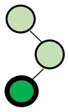 **Man3**	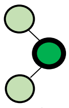 **Manβ**
H1/C1	5.19/98.5	5.19/98.5	4.92/99.3	4.92/99.3	5.20/99.6	4.83/100.5
H2/C2	3.76/67.8	3.76/67.8	3.86/68.3	3.82/68.2	4.25/76.6	4.308/70.3
H3/C3	4.00/69.3	4.00/69.3	3.91/69.6	3.91/69.6	3.96/69.5	3.84/80.5
H4/C4	3.90/72.1	3.85/72.1	3.84/71.9	3.84/71.9	3.56/67.4	3.86/65.8
H5/C5	4.91/66.9	4.88/66.7	4.14/66.8	4,08/66.8	3.80/73.6	3.67/74.6
H6/C6	1.31/15.5	1.23/15.4	1.25/15.6	1.19/15.5	3.68, 3.96/61.8	4.02, 3.87/66.0

	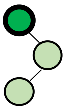 **Man6**	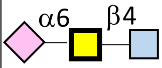 **GalNAc (2,6Neu5Ac)**	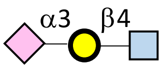 **Gal (2,3Neu5Ac)**	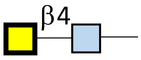 **GalNAc (LDN)**	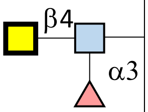 **GalNAc (LDNF)**	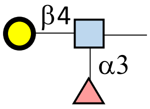 **Gal (Le^{x})**
H1/C1	4.99/97.2	4.57/102.3	4.52/103.6	4.59/101.9	4.53/100.8	4.51/101.9
H2/C2	4.17/76.5	4.00/52.6	3.60/70.9	4.01/52.5	4.02/52.6	3.55/71.2
H3/C3	3.95/69.5	3.82/70.8	3.73/72.6	3.83/70.7	3.79/70.8	3.71/72.7
H4/C4	3.55/67.5	4.03/67.5	4.00/68.6	4.01/67.7	3.97/67.5	3.97/68.4
H5/C5	3.68/72.9	3.89/73.8	3.89/73.84	nd	3.64/74.9	3.65/75.0
H6/C6	3.68, 3.96/61.8	4.08, 3.64/63.5	4.05, 3.64/63.5	3.81/61.00	3.80/61.5	3.81/61.6

	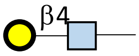 **Gal terminal**	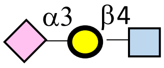 **Gal (2,3Neu5Ac)**	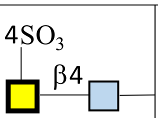 **4SO3-GalNAc**	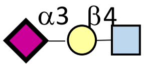 **Neu5Ac α2-3**	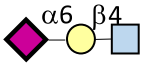 **Neu5Ac α2-6**	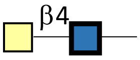 **GlcNAc (b1-4GlcNAc)**
H1/C1	4.54/103.1	4.61/102.8	4.67/101.8			4.62/101.7
H2/C2	3.61/71.2	3.64/69.5	3.99/53.0			3.80/55.1
H3/C3	3.73/72.7	4.18/75.6	3.98/69.8	2.82, 1.85/39.7	2.73, 1.76/40.2	3.79/72.4
H4/C4	4.00/68.6	4.03/67.6	4.76/75.6	3.77/68.5	3.73/68.3	3.80/78.7
H5/C5	3.80/75.5	3.79/75.3	3.9/74.7	3.91/51.9	3.87/52.0	nd
H6/C6	3.81/61.1	3.81/61.1	3.83/61.0	3.72/73.0	3.77/72.7	4.06, 3.74/60.2
H7/C7				3.66/68.5	3.63/68.5	
H8/C8				3.96/71.9	3.96/71.9	
H9/C9				3.94, 3.72/62.8	3.94, 3.72/62.8	

6. Figure S2

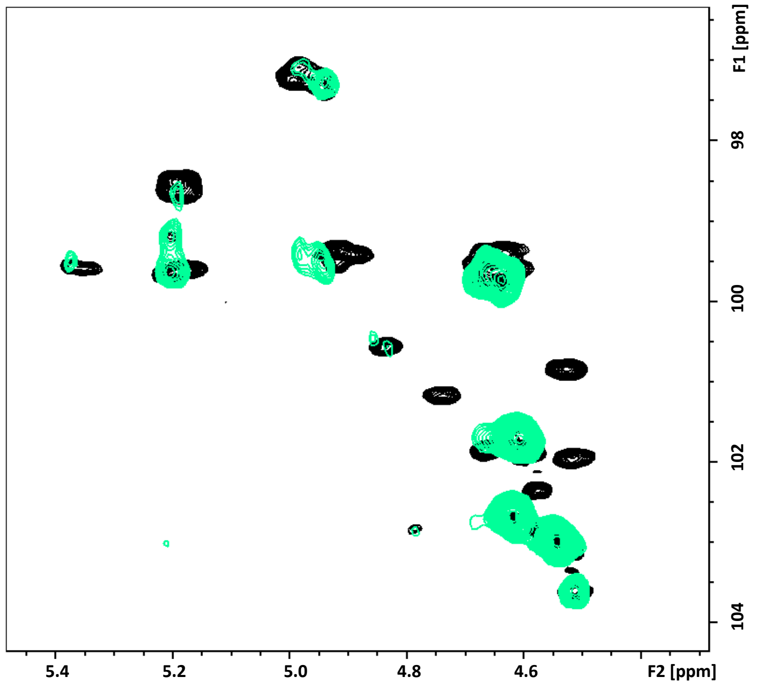

Figure S2. Superimposition of the $^{1}H,^{13}C$-HSQC spectra, at the anomeric region, of the two glycoproteins produced in HEK293 as described above. Black: RBD (amino acid residues 328-533) from the spike protein of SARS-CoV-2. Green: FcεRIα.

7. Figure S3

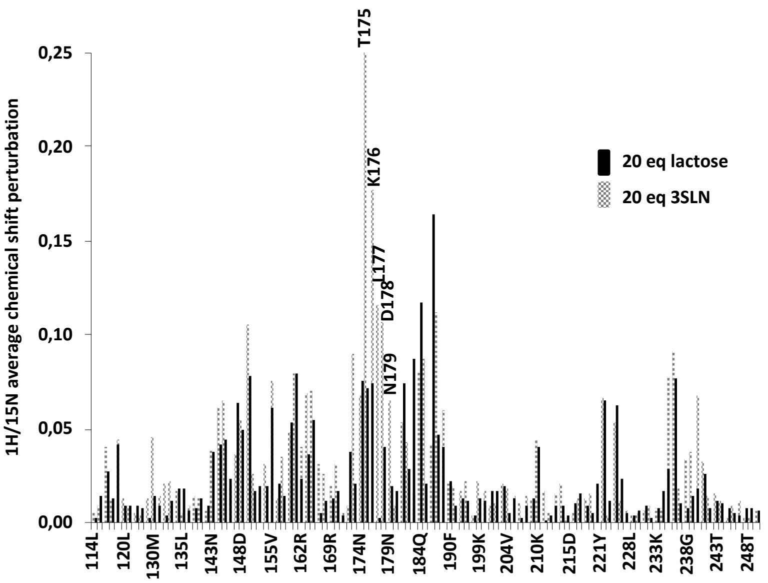

Figure S3. Average chemical shift perturbation (Δ_{av}) of ^{15}N-galectin-3 CRD (on $^{1}H,^{15}N$-TROSY) produced by the addition of 20 equivalents of lactose and 20 equivalents of 3´SLN trisaccharide. $\Delta_{av} = [(\Delta\delta(_{NH})^2 + (\Delta\delta(_{N})/5)^2]^{1/2}$.

8. Figure S4

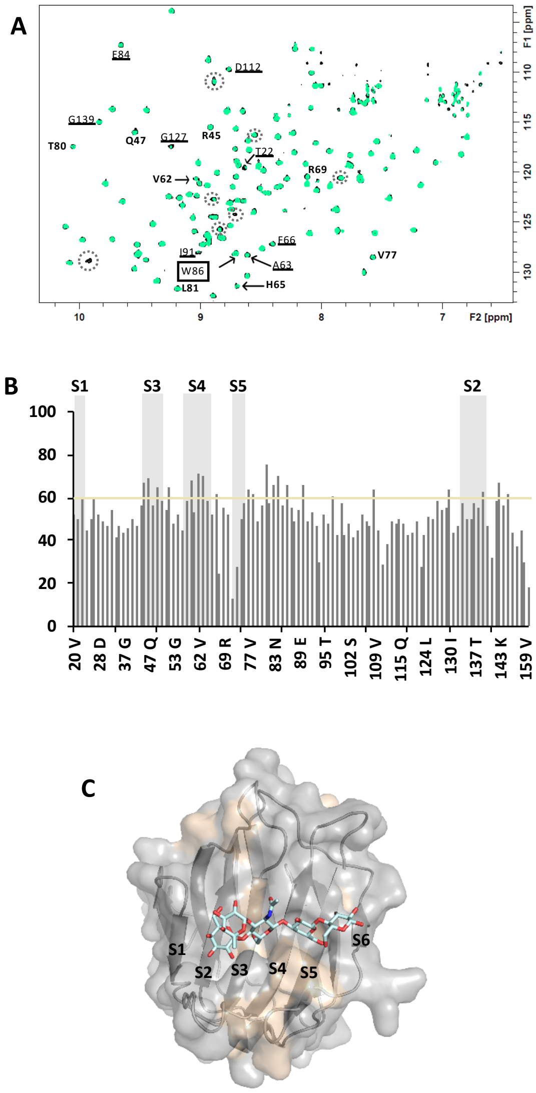

Figure S4. The interaction between galectin-8N and the RBD as deduced by NMR from the lectin perspective. A. Overlay of the $^{1}H,^{15}N$-TROSY spectra of galectin-8N (black) and in the presence of 0.4 equivalents of RBD (green). Broken lines indicate not assigned cross peaks. In bold, residues at the canonical glycan binding site and underlined, residues far from the binding site. The conserved Trp at the glycan binding site (W86) is squared. B. Graphical bar representation of the % cross peak volume reduction on the $^{1}H,^{15}N$-TROSY spectrum of galectin-8N upon the addition of 0.4 equivalents of RBD. S1-S6 β-strands are depicted with grey boxes. The wheat horizontal line indicates the NMR cross peaks suffering more than a 60 % of signal reduction. C. Cartoon and surface representation of galectin-8N complexed with 3'SLN (PDB ID 5G7F) according to the crystal structure. Amino acids are coloured based on their perturbation (% cross peak volume reduction) due to the presence of the RBD according to the threshold established in B.

9. Figure S5

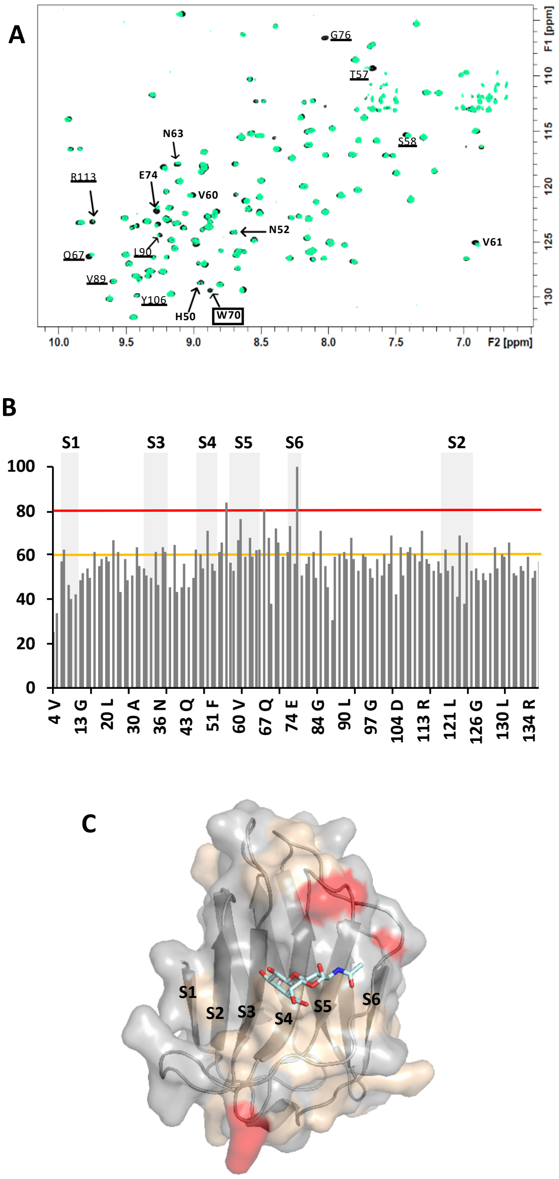

Figure S5. Interaction between galectin-7 and the RBD as deduced by NMR from the lectin perspective. A. Superposition of the ^{1}H,^{15}N-TROSY spectra of galectin-7 (black) and in the presence of 1 equivalent of RBD (green). In bold, residues at the canonical glycan binding site and underlined, residues far from the binding site. The conserved Trp at the glycan binding site (W70) is squared. B. Graphical bar representation of the % cross peak volume reduction on the ^{1}H,^{15}N-TROSY spectrum of galectin-7 upon the addition of 1 equivalent of RBD. S1-S6 β-strands are depicted with grey boxes. The red and wheat horizontal lines, are baselines for NMR cross peaks suffering 80-100% signal reduction and 60-80% signal reduction, respectively. C. Cartoon and surface representation of galectin-7 complexed with LacNAc (PDB ID 5GAL) according to the crystal structure. Amino acids are coloured based on their perturbation (% cross peak volume reduction) due to the presence of the RBD according to the thresholds established in B.

10. Figure S6

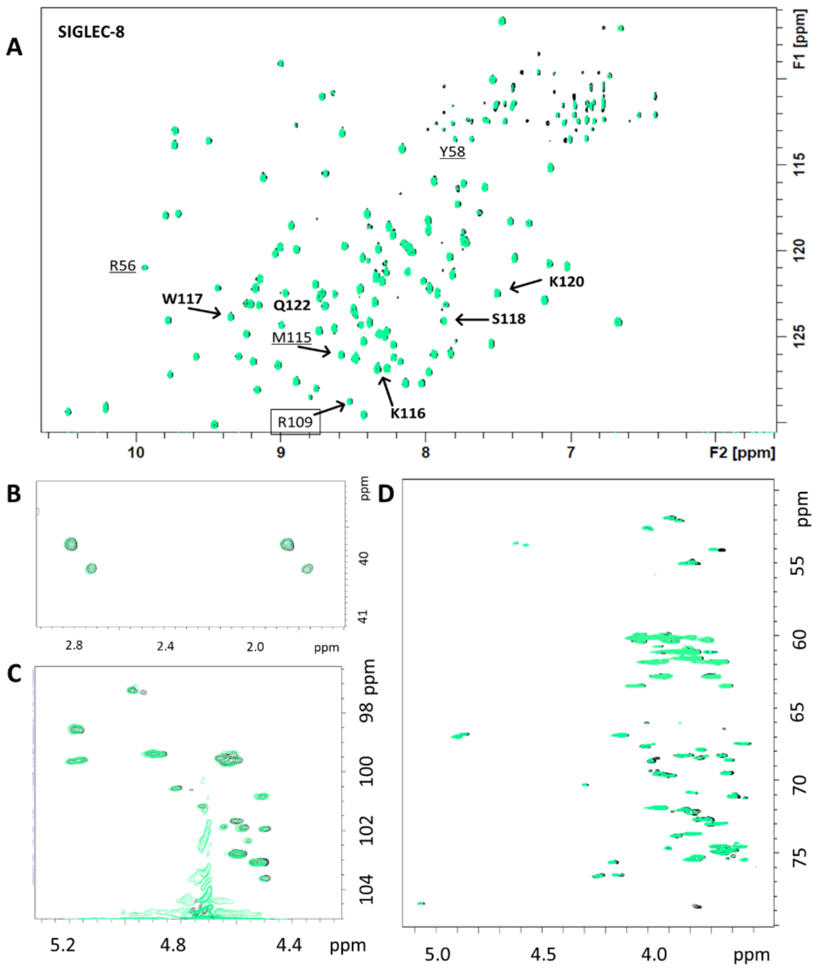

Figure S6. Siglec-8 does not interact with RBD, as deduced by NMR both from the lectin perspective and the glycans perspective. A. Superposition of the $^{1}H,^{15}N$-TROSY spectra of siglec-8 (black) alone and in the presence of 1 equivalent of RBD (green). Some cross peaks are annotated. B-D. Different regions of the superimposition of the $^{1}H,^{13}C$-HSQC spectra of RBD with ^{13}C-glycans in the absence (black) and in the presence of Siglec-8 (green).

11. Figure S7

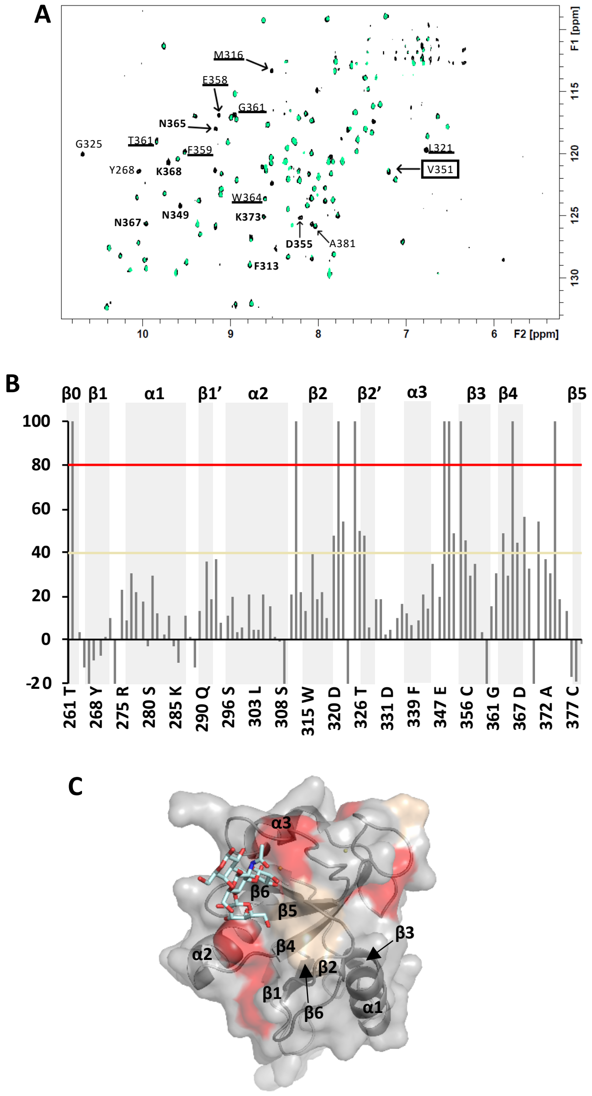

Figure S7. Interaction between DC-SIGN CRD and the RBD as deduced by NMR from the lectin perspective. A. Superposition of the ^{1}H,^{15}N-TROSY spectra of DC-SIGN (black) and in the presence of 1 equivalent of RBD (green). In bold, residues at the canonical glycan binding site and underlined, residues far from the binding site. The V351 residue at the edge of the long loop at the primary Ca^{+2} binding site is squared. B. Graphical bar representation of the % cross peak volume reduction on the ^{1}H,^{15}N-TROSY spectrum of DC-SIGN upon the addition of RBD. β-strands and helices are depicted with grey boxes. The red and wheat horizontal lines, are baselines for NMR cross peaks suffering 80-100% signal reduction and 40-80% signal reduction, respectively. C. Cartoon and surface representation of DC-SIGN complexed with LeX (PDB ID 1SL5) according to the crystal structure. Amino acids are coloured based on their perturbation (% cross peak volume reduction) due to the presence of the RBD according to the thresholds established in B.

12. Figure S8

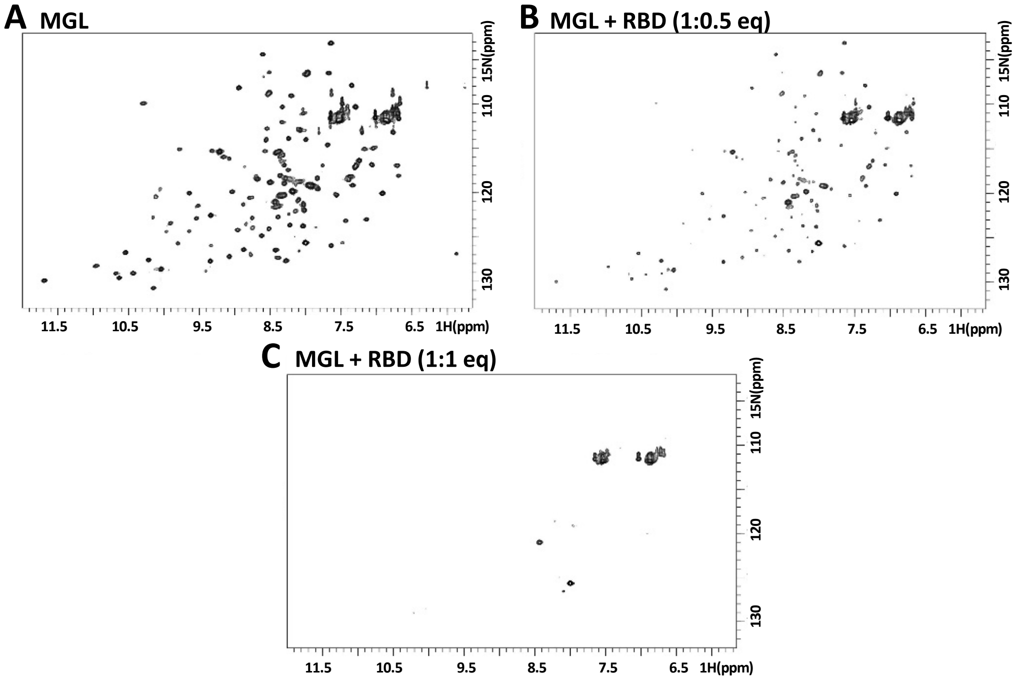

Figure S8. A) $^{1}H,^{15}N$-HSQC spectrum of MGL CRD. B) $^{1}H,^{15}N$-HSQC spectrum of MGL CRD and 0.5 equivalent of RBD. C) $^{1}H,^{15}N$-HSQC spectrum of MGL CRD and 1 equivalent of RBD.

13. Figure S9

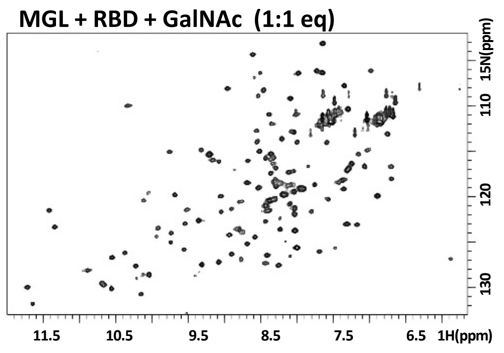

Figure S9. Competition experiment. $^{1}H,^{15}N$-HSQC spectrum of MGL CRD with 1 equivalent of RBD and 1 equivalent of GalNAc. The observed spectrum is identical to the $^{1}H,^{15}N$-HSQC of MGL-CRD in presence of GalNAc previously reported in Diniz et al.[7]

14. Figure S10.

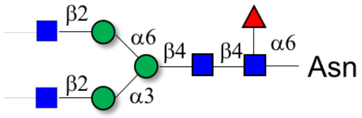

Figure S10. Glycan core common to all RBD biantennary glycans modelled in this study.

15. Table S2

Table S2. Antennae glycan composition of the glycosylated RBD models.

Model	Receptor	N-glycosylation position	Glycan
Gal3_RBD-N331-3SLacNAc	Galectin-3	N331	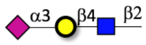 3SLacNAc
Gal3_RBD-N343-3SLacNAc		N343	
Gal7_RBD-N331-LDN	Galectin-7	N331	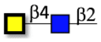 LDN
Gal7_RBD-N343-LDN		N343	
Gal8_RBD-N331-3SLacNAc	Galectin-8 N term	N331	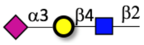 3SLacNAc
Gal8_RBD-N343-3SLacNAc		N343	
DC-SIGN_RBD-N331-LeX	DC-SIGN	N331	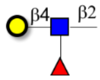 LeX
DC-SIGN_RBD-N343-LeX		N343	
MGL_RBD-N331-6SLDN	MGL	N331	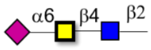 6SLDN
MGL_RBD-N343-6SLDN		N343	

16. Figure S11

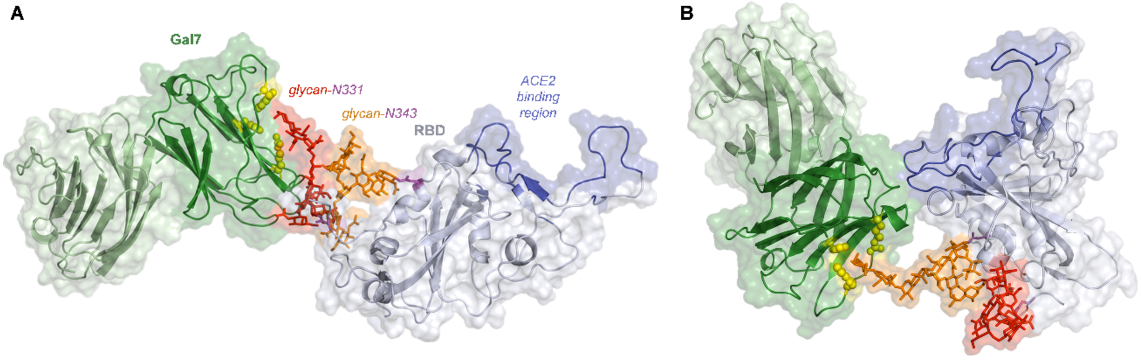

Figure S11. Molecular models for the 1:1 complexes of galectin-7 (PDB ID 4GAL) with the LDN epitope attached at both glycosylation sites of the RBD of SARS CoV-2, according to selected snapshots of 200 ns MD simulations (A,B: binding through glycans at positions N331 and N343, respectively). Galectin-7 and RBD are shown as green and grey cartoons, respectively. The ACE2 recognition of RBD is shown in blue. Glycans at glycosylation sites N331 and N343 are shown as red and orange sticks, respectively. RBD glycosylated N residues are shown as magenta sticks. Binding site residues H50, R54 and W70 in galectin-7 are shown as yellow spheres. All hydrogens have been omitted for clarity.

17. Figure S12.

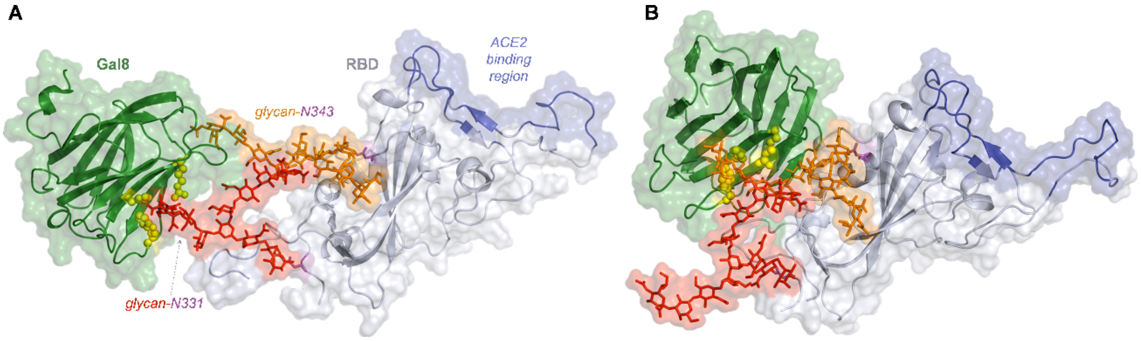

Figure S12. Molecular models for the 1:1 complexes of galectin-8 N-terminal (PDB ID 5G7F) with the 3'SLacNAc epitope attached at both glycosylation sites of the RBD of SARS CoV-2, according to selected snapshots of 200 ns MD simulations (A,B: binding through glycans at positions N331 and N343, respectively). Galectin-8 N-terminal CRD and RBD are shown as green and grey cartoons, respectively. The ACE2 recognition of RBD is shown in blue. Glycans at glycosylation sites N331 and N343 are shown as red and orange sticks, respectively. RBD glycosylated N residues are shown as magenta sticks. Binding site residues H65, R69 and W86 in galectin-8 N-terminal CRD are shown as yellow spheres. All hydrogens have been omitted for clarity.

18. Figure S13

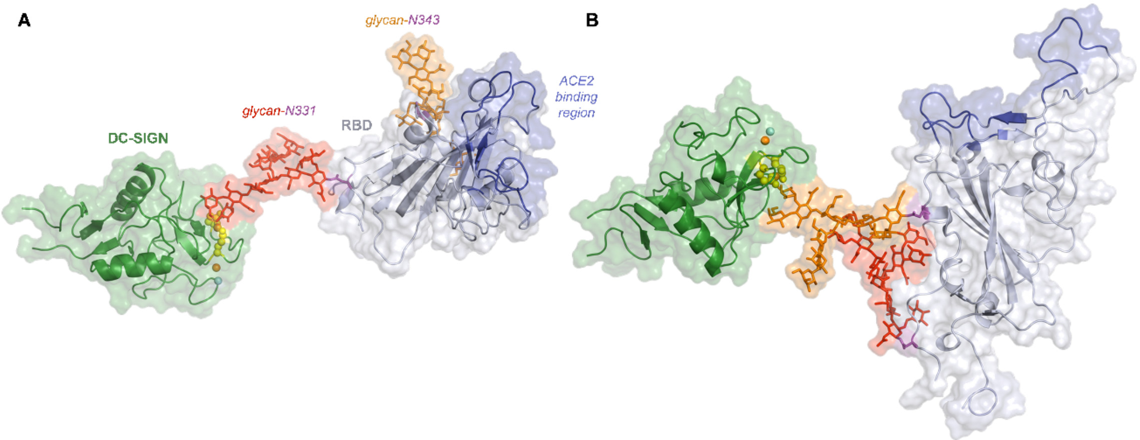

Figure S13. Molecular models for the 1:1 complexes of DC-SIGN CRD (PDB ID 1SL5) with the LeX epitope attached at both glycosylation sites of the RBD of SARS CoV-2, according to selected snapshots of 200 ns MD simulations (A,B: binding through glycans at positions N331 and N343, respectively). DC-SIGN CRD and RBD are shown as green and grey cartoons, respectively. The ACE2 recognition of RBD is shown in blue. Glycans at glycosylation sites N331 and N343 are shown as red and orange sticks, respectively. RBD glycosylated N residues are shown as magenta sticks. Binding site calcium atom and residues E347 and E354 in DC-SIGN CRD are shown as orange and yellow spheres, respectively. All hydrogens have been omitted for clarity.

19. Figure S14.

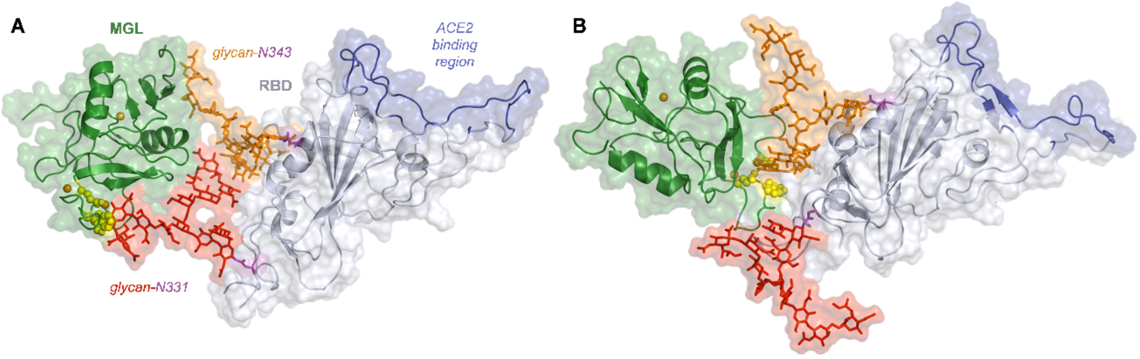

Figure S14. Molecular models for the 1:1 complexes of MGL CRD with the 6SLDN epitope attached at both glycosylation sites of the RBD of SARS CoV-2, according to selected snapshots of 200 ns MD simulations (A,B: binding through glycans at positions N331 and N343, respectively). MGL CRD and RBD are shown as green and grey cartoons, respectively. The ACE2 recognition of RBD is shown in blue. Glycans at glycosylation sites N331 and N343 are shown as red and orange sticks, respectively. RBD glycosylated N residues are shown as magenta sticks. Binding site calcium atom and interacting residues in MGL CRD are shown as orange and yellow spheres, respectively. All hydrogens have been omitted for clarity.

20. Table S3.

Table S3. List of videos (MP4 format) and PyMol sessions (version 2.0 or later needed) depicting 200 ns MD simulations for the 3D models generated in this study.

**Videos**	**Pymol sessions**

Gal3_RBD-N331-3SLAcN.mp4	Gal3_RBD-N331-3SLAcN.pse
Gal3_RBD-N343-3SLAcN.mp4	Gal3_RBD-N343-3SLAcN.pse
Gal7_RBD-N331-LDN.mp4	Gal7_RBD-N331-LDN.pse
Gal7_RBD-N343-LDN.mp4	Gal7_RBD-N343-LDN.pse
Gal8_RBD-N331-3SLacN.mp4	Gal8_RBD-N331-3SLacN.pse
Gal8_RBD-N343-3SLacN.mp4	Gal8_RBD-N343-3SLacN.pse
DC-SIGN_RBD-N331-LeX.mp4	DC-SIGN_RBD-N331-LeX.pse
DC-SIGN_RBD-N343-LeX.mp4	DC-SIGN_RBD-N343-LeX.pse
MGL_RBD-N331-6SLDN.mp4	MGL_RBD-N331-6SLDN.pse
MGL_RBD-N343-6SLDN.mp4	MGL_RBD-N343-6SLDN.pse

21. Bibliography

[1] A. R. Aricescu, W. Lu, E. Y. Jones, *Acta Crystallogr. Sect. D Biol. Crystallogr.* **2006**, *62*, 1243–1250.

[2] A. W. Barb, D. J. Falconer, G. P. Subedi, in *Methods Enzymol.*, Academic Press Inc., **2019**, pp. 239–261.

[3] H. Yagi, Y. Zhang, M. Yagi-Utsumi, T. Yamaguchi, S. Iida, Y. Yamaguchi, K. Kato, *Biomol. NMR Assign.* **2015**, *9*, 257–260.

[4] L. Unione, M. P. Lenza, A. Ardá, P. Urquiza, A. Laín, J. M. Falcón-Pérez, J. Jiménez-Barbero, O. Millet, *ACS Cent. Sci.* **2019**, *5*, 1554–1561.

[5] A. Gimeno, S. Delgado, P. Valverde, S. Bertuzzi, M. A. Berbis, J. Echavarren, A. Lacetera, S. Martin-Santamaria, A. Surolia, F. J. Canada, J. Jimenez-Barbero, A. Arda, *Angew. Chem. Int. Ed. Engl.* **2019**, *58*, 7268–7272.

[6] J. M. Propster, F. Yang, S. Rabbani, B. Ernst, F. H.-T. Allain, M. Schubert, *Proc. Natl. Acad. Sci. U. S. A.* **2016**, *113*, E4170-9.

[7] A. Diniz, H. Coelho, J. S. Dias, S. J. van Vliet, J. Jiménez-Barbero, F. Corzana, E. J. Cabrita, F. Marcelo, *Chem. - A Eur. J.* **2019**, *25*, 13945–13955.

[8] M. S. Rana, X. Wang, A. Banerjee, *Biochemistry* **2018**, *57*, 6741–6751.

[9] S. W. Fesik, H. L. Eaton, E. T. Olejniczak, E. R. P. Zuiderweg, L. P. McIntosh, F. W. Dahlquist, *J. Am. Chem. Soc.* **1990**, *112*, 886–888.

[10] L. E. Kay, G. Y. Xu, A. U. Singer, D. R. Muhandiram, J. D. Formankay, *J. Magn. Reson. Ser. B* **1993**, *101*, 333–337.

[11] T. M. Logan, E. T. Olejniczak, R. X. Xu, S. W. Fesik, *J. Biomol. NMR* **1993**, *3*, 225–231.

[12] K. Umemoto, H. Leffler, *J. Biomol. NMR* **2001**, 91–92.

[13] I. V Nesmelova, M. Á. Berbís, M. C. Miller, F. J. Cañada, S. André, J. Jiménez-Barbero, H.-J. Gabius, K. H. Mayo, *Biomol. NMR Assign.* **2012**, *6*, 127–129.

[14] P. Valverde, J. D. Martínez, F. J. Cañada, A. Ardá, J. Jiménez-Barbero, *ChemBioChem* **2020**, *21*, 1–28.

[15] J. M. Pröpster, F. Yang, S. Rabbani, B. Ernst, F. H. T. Allain, M. Schubert, *Proc. Natl. Acad. Sci.* **2016**, *113*, E4170–E4179.

[16] W. F. Vranken, W. Boucher, T. J. Stevens, R. H. Fogh, A. Pajon, M. Llinas, E. L. Ulrich, J. L. Markley, J. Ionides, E. D. Laue, *Proteins Struct. Funct. Bioinforma.* **2005**, *59*, 687–696.

[17] J. Su, T. Zhang, P. Wang, F. Liu, G. Tai, Y. Zhou, *Acta Biochim. Biophys. Sin. (Shanghai).* **2015**, *47*, 192–198.

[18] D. D. Leonidas, E. H. Vatzaki, H. Vorum, J. E. Celis, P. Madsen, K. R. Acharya, *Biochemistry* **1998**, *37*, 13930–13940.

[19] Y. Si, Y. Wang, J. Gao, C. Song, S. Feng, Y. Zhou, G. Tai, J. Su, *Int. J. Mol. Sci.* **2016**, DOI 10.3390/ijms17122088.

[20] Y. Guo, H. Feinberg, E. Conroy, D. A. Mitchell, R. Alvarez, O. Blixt, M. E. Taylor, W. I. Weis, K. Drickamer, *Nat. Struct. Mol. Biol.* **2004**, *11*, 591–598.

[21] C. A. Sanhueza, M. M. Baksh, B. Thuma, M. D. Roy, S. Dutta, C. Préville, B. A. Chrunyk, K. Beaumont, R. Dullea, M. Ammirati, S. Liu, D. Gebhard, J. E. Finley, C. T. Salatto, A. King-Ahmad, I. Stock, K. Atkinson, B. Reidich, W. Lin, R. Kumar, M. Tu, E. Menhaji-Klotz, D. A. Price, S. Liras, M. G. Finn, V. Mascitti, *J. Am. Chem. Soc.* **2017**, *139*, 3528–3536.

[22] Y. Song, F. DiMaio, R. Y.-R. Wang, D. Kim, C. Miles, T. Brunette, J. Thompson, D. Baker, *Structure* **2013**, *21*, 1735–1742.

[23] D. Wrapp, N. Wang, K. S. Corbett, J. A. Goldsmith, C.-L. Hsieh, O. Abiona, B. S. Graham, J. S. McLellan, *Science (80-.).* **2020**, *367*, 1260–1263.

[24] D. Wrapp, N. Wang, K. S. Corbett, J. A. Goldsmith, C.-L. Hsieh, O. Abiona, B. S. Graham, J. S. McLellan, *Science (80-.).* **2020**, *367*, 1260–1263.

[25] H. Woo, S.-J. Park, Y. K. Choi, T. Park, M. Tanveer, Y. Cao, N. R. Kern, J. Lee, M. S. Yeom, T. I. Croll, C. Seok, W. Im, *J. Phys. Chem. B* **2020**, acs.jpcb.0c04553.

[26] S. Jo, T. Kim, V. G. Iyer, W. Im, *J. Comput. Chem.* **2008**, *29*, 1859–1865.

[27] W. L. DeLano, V. 2. . The PyMOL Molecular Graphics System, Schrödinger LLC 2020

[28] Woods Group. (2005-2020) GLYCAM Web. Complex Carbohydrate Research Center, University of Georgia, Athens, GA. (http://glycam.org)

[29] D. A. Case, I. Y. Ben-Shalom, S. R. Brozell, D. S. Cerutti, T. E. Cheatham, III, V. W. D. Cruzeiro, T. A. Darden, R. E. Duke, D. Ghoreishi, M. K. Gilson, H. Gohlke, A. W. Goetz, D. Greene, R. Harris, N. Homeyer, Y. Huang, S. Izadi, A. Kovalenko, T. Kurtzman, T. S. Lee, S. LeGrand, P. Li, C. Lin, J. Liu, T. Luchko, R. Luo, D. J. Mermelstein, K. M. Merz, Y. Miao, G. Monard, C. Nguyen, H. Nguyen, I. Omelyan, A. Onufriev, F. Pan, R. Qi, D. R. Roe, A. Roitberg, C. Sagui, S. Schott-Verdugo, J. Shen, C. L. Simmerling, J. Smith, R. S.- Ferrer, J. Swails, R. C. Walker, J. Wang, H. Wei, R. M. Wolf, X. Wu, L. Xiao, D. M. York, P. A. Kollman, *Amber 2018*, **2018**, University of California San Francisco.

[30] T.-S. Lee, D. S. Cerutti, D. Mermelstein, C. Lin, S. LeGrand, T. J. Giese, A. Roitberg, D. A. Case, R. C. Walker, D. M. York, *J. Chem. Inf. Model.* **2018**, *58*, 2043–2050.

[31] J. A. Maier, C. Martinez, K. Kasavajhala, L. Wickstrom, K. E. Hauser, C. Simmerling, *J. Chem. Theory Comput.* **2015**, *11*, 3696–3713.

[32] K. N. Kirschner, A. B. Yongye, S. M. Tschampel, J. González-Outeiriño, C. R. Daniels, B. L. Foley, R. J. Woods, *J. Comput. Chem.* **2008**, *29*, 622–655.

[33] W. L. Jorgensen, J. Chandrasekhar, J. D. Madura, R. W. Impey, M. L. Klein, *J. Chem. Phys.* **1983**, *79*, 926–935.

[34] H. C. Andersen, *J. Chem. Phys.* **1980**, *72*, 2384–2393.

[35] T. A. Andrea, W. C. Swope, H. C. Andersen, *J. Chem. Phys.* **1983**, *79*, 4576–4584.

[36] S. Miyamoto, P. A. Kollman, *J. Comput. Chem.* **1992**, *13*, 952–962.

[37] T. Darden, D. York, L. Pedersen, *J. Chem. Phys.* **1993**, *98*, 10089–10092.
